# Benthic invertebrates in Svalbard fjords—when metabarcoding does not outperform traditional biodiversity assessment

**DOI:** 10.7717/peerj.14321

**Published:** 2022-11-17

**Authors:** Endre Willassen, Jon-Ivar Westgaard, Jon Anders Kongsrud, Tanja Hanebrekke, Pål Buhl-Mortensen, Børge Holte

**Affiliations:** 1 Department of Natural History, University of Bergen, Bergen, Norway; 2 Department of Population Genetics, Institute of Marine Research, Tromsø, Troms, Norway; 3 Department of Bentic Communities, Institute of Marine Research, Bergen, Norway; 4 Department of Bentic Communities, Institute of Marine Research, Tromsø, Troms, Norway

**Keywords:** eDNA, Metabarcoding, Invertebrates, Taxonomy, Marine sediments

## Abstract

To protect and restore ecosystems and biodiversity is one of the 10 challenges identified by the United Nations’s Decade of the Ocean Science. In this study we used eDNA from sediments collected in two fjords of the Svalbard archipelago and compared the taxonomic composition with traditional methods through metabarcoding, targeting mitochondrial CO1, to survey benthos. Clustering of 21.6 mill sequence reads with a d value of 13 in *swarm*, returned about 25 K OTU reads. An identification search with the BOLD database returned 12,000 taxonomy annotated sequences spanning a similarity range of 50% to 100%. Using an acceptance filter of minimum 90% similarity to the CO1 reference sequence, we found that 74% of the ca 100 taxon identified sequence reads were Polychaeta and 22% Nematoda. Relatively few other benthic invertebrate species were detected. Many of the identified sequence reads were extra-organismal DNA from terrestrial, planktonic, and photic zone sources. For the species rich Polychaeta, we found that, on average, only 20.6% of the species identified from morphology were also detected with DNA. This discrepancy was not due to missing reference sequences in the search database, because 90–100% (mean 96.7%) of the visually identified species at each station were represented with barcodes in Boldsystems. The volume of DNA samples is small compared with the volume searched in visual sorting, and the replicate DNA-samples in sum covered only about 2% of the surface area of a grab. This may considerably reduce the detection rate of species that are not uniformly distributed in the sediments. Along with PCR amplification bias and primer mismatch, this may be an important reason for the limited congruence of species identified with the two approaches. However, metabarcoding also identified 69 additional species that are usually overlooked in visual sample sorting, demonstrating how metabarcoding can complement traditional methodology by detecting additional, less conspicuous groups of organisms.

## Introduction

The United Nation’s Decade of Ocean Science has highlighted 10 challenges, one of which is to protect and restore ecosystems and biodiversity. Adequate measures towards such aims certainly require fundamental knowledge about the units of biodiversity, their interaction with the environment, and how to assess whether an ecosystem should be considered naturally healthy or disturbed by unwanted anthropogenic impacts. Bioassessment and monitoring is usually based on species identification and evaluation of ecological conditions as indicated by species communities in the target habitat. Historically, species identifications rely on visual examination of morphological characters and taxonomic decisions based on established diagnostic characters. With increasing demands for biodiversity data and a depauperate population of researchers able to satisfy the demands, traditional identification procedures have been regarded as a processing bottleneck caused by a so-called “taxonomic impediment” (*e.g.*, Tautz et al., 2003), and identification with DNA-barcodes (Hebert et al., 2003) been used as efficient alternatives to traditional methods. The technological developments of high throughput DNA sequencing have offered new tools to study biodiversity based on environmental DNA (eDNA) (Taberlet et al., 2012a, 2012b; Ji et al., 2013; Gibson et al., 2015; Lacoursière-Roussel et al., 2018; Wangensteen & Turon, 2017; Schadewell & Adams, 2021; Bohmann et al., 2021; Mugnai et al., 2021). Whilst some applications in marine environments have been relatively open-end inventories of metazoan diversity (*e.g.*, Leray & Knowlton, 2016), metabarcoding, which enables a simultaneous detection of several taxa within the same sample, has also been promoted as a technological advance that may either complement or even supersede traditional methods for biological monitoring and management (Aylagas, Borja & Rodríguez-Ezpeleta, 2014; Aylagas et al., 2016, 2018; Creer et al., 2016; Gold et al., 2021; Descôteaux et al., 2021). From the perspective of management and conservation, the purpose of eDNA studies may be to detect and monitor specific selected taxa that are considered either invasive and unwanted or elusive and endangered (*e.g.*, Biggs et al., 2015; Wilcox et al., 2013; Duarte et al., 2021; Ibabe et al., 2021). Multispecies metabarcoding studies have broader perspectives on biodiversity, aiming to infer species composition, ecological communities, trophic relationships, food webs or other ecological interaction. Such studies may be more challenging than single species studies for several reasons, some of which we will highlight in this article. These challenges are certainly also relevant for environmental assessment and monitoring of anthropogenic impacts.

Some approaches to assess the ecological state of target environments do not rely on taxonomic identification of the interacting types of organisms in the system and are accordingly called “taxon-free” (*e.g.*, Pawlowski et al., 2018, 2021; Mächler, Walser & Altermatt, 2020). Although unidentified Operational Taxonomic Units (OTUs) allow for calculation of biodiversity statistics that could be used to evaluate ecological conditions in an environment, most empirical understanding of relationship between environment and biodiversity is deeply rooted in taxonomic identification of detected units in the system and (some minimum) of associated knowledge about their distributions, ecological function, habitat preferences and tolerances to impacts from natural and environmental factors. The paucity of such knowledge is one disadvantage of taxon-free approaches. Another is that sequence similarity based OTUs in each dataset are context dependent and difficult to compare with OTUs from other datasets unless they can be referred to a labelled-OTU reference library (Callahan, McMurdie & Holmes, 2017; Pappalardo et al., 2021), although this issue can be overcome by using exact sequence variants, or ESVs (Callahan, McMurdie & Holmes, 2017; Porter & Hajibabaei, 2020).

Thus, taxon-based approaches hold more promise of cohesive terminology, external consistency of taxonomies, repeatability, and links to traditional empirical knowledge about the individual taxa encountered in a study system. However, although DNA metabarcoding can theoretically “assign taxonomy to hundreds of samples rapidly and at low cost” (Aylagas et al., 2018), such procedures rely on good quality sequence databases (Ekrem, Stuhr & Willassen, 2007) and studies have shown that relatively moderate fractions of the marine species diversity have been assigned with DNA barcode markers (McGee, Robinson & Hajibabaei, 2019; Wangensteen et al., 2018; Günther et al., 2018, 2019; Weigand et al., 2019; Hestetun et al., 2020; Mugnai et al., 2021). In addition, eDNA-based species inventories have some particular challenges related to “false positive” and “false negative” observations (Rees et al., 2014; Cowart et al., 2015; Ficetola et al., 2014, 2015; Roussel et al., 2015; McClenaghan, Compson & Hajibabaei, 2020). When identifying an eDNA sequence based on similarity with an annotated reference sequence, a false positive may result if the reference sequence is misidentified, or if the marker is actually unable to discriminate between taxa at the reported hierarchical level. A special class of false positives are observations that stem from either contamination introduced with sample handling or from extraneous DNA depositions. Epistemologically, such mistakes can be considered as “type I errors”. In this article, we will provide examples to highlight the importance a validated taxonomy in a Supplemental Text (Supplement S1). False negatives are target entities that were undetected, despite being present in the observation field. The problem with false negatives is accentuated in gaps between morphologically identified taxa and missing detections from eDNA. Such incongruent observations may have different causes.

Despite such challenges, one obvious advantage of identifications based on reference databases is that they will help obtaining unified understanding of biodiversity units and a standardized taxonomy across regional scientific cultures where a species may go by different names, or the same name is used for different species. This is particularly important for conservation measures (Bortolus, 2008) and when comparing biodiversity investigations across regional scientific research (*e.g.*, Teixeira et al., 2016; Uusitalo et al., 2016). Efforts for such unification of taxonomies are amongst the recommendations from the Conservation of Arctic Flora and Fauna consortium (CAFF, 2013, 2017).

In this article we examine the species composition of soft bottom communities sampled with benthic grab in two Arctic fjords to explore the comparability between metabarcoding and morphology-based species identifications. This study was initiated by the Norwegian Mareano seafloor mapping programme (www.mareano.no; Buhl-Mortensen et al., 2015) to investigate whether metabarcoding may represent a cost-effective species identification alternative without serious deviations from the ongoing morphological identification and also to develop metabarcoding as a benthic species identification tool in baseline and monitoring environmental exercises. We therefore compare metazoan biodiversity with the two approaches, and explore, with indirect analyses, if any differences in relationships between community composition and MAREANO-standard environmental parameters are indicated from the two sets of taxa-data.

The Mareano collections have provided considerable contributions to the diversity of marine species barcoded in the Norwegian Barcode of Life programme (NORBOL, https://www.norbol.org/), and helped to prepare the ground for DNA-based identification, particularly of North Atlantic and Arctic benthic fauna. The voucher material for these is kept in the University Museum of Bergen (*e.g.*, Willassen et al., 2019) and as opposed to a statement in Mugnai et al. (2021), the barcode data from the NORBOL consortium are still being curated and under expansion with more barcodes of marine biodiversity.

## Methods

### Stations and sampling

**Figure 1:**
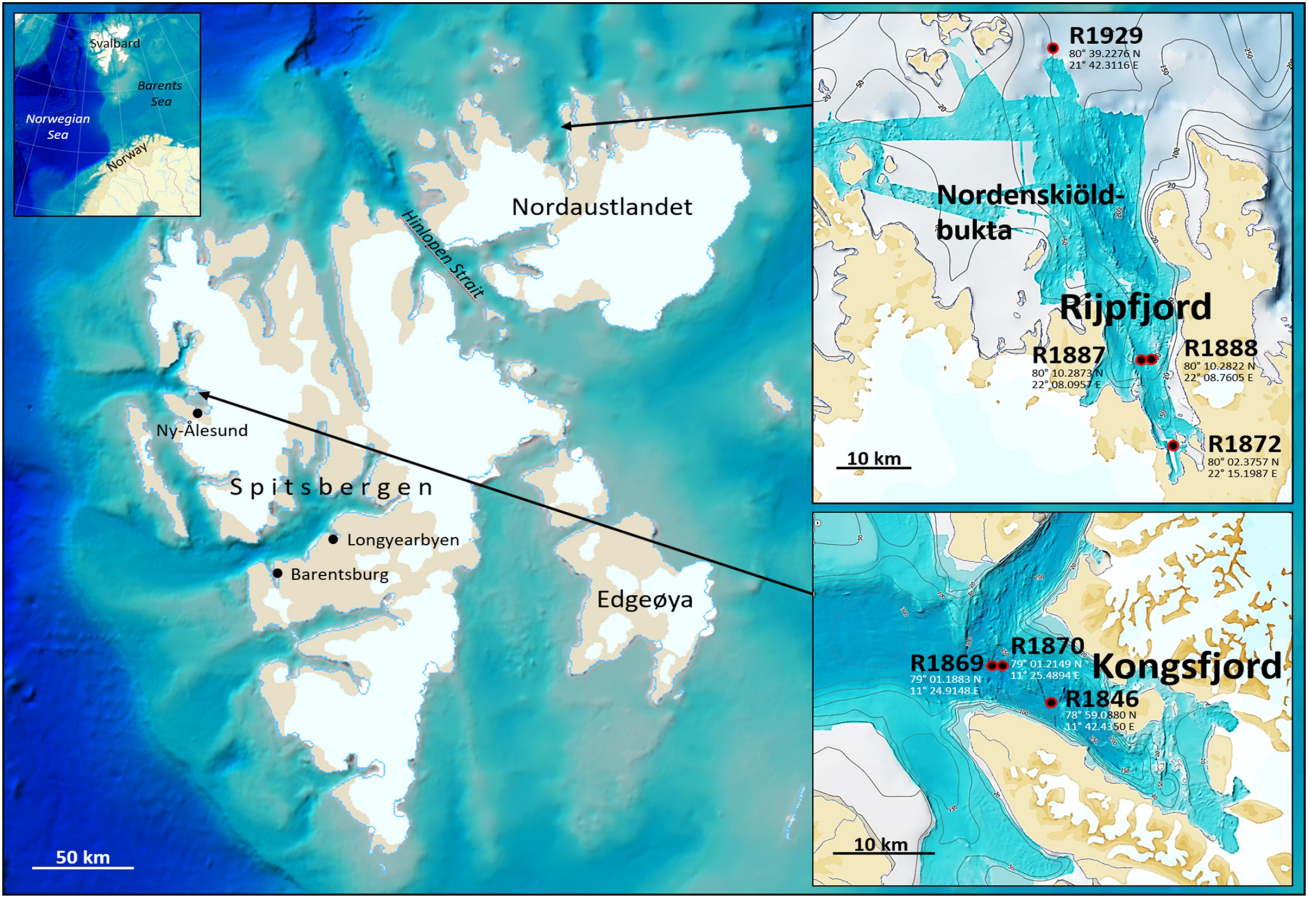
eDNA sampling stations in Rijpfjord and Kongsfjord, Svalbard. GEBCO large-scale background maps. Detailed topography maps from www.mareano.no.

Fauna- and eDNA samples were collected at three stations in Kongsfjord and four stations in Rijpfjord (Fig. 1), Svalbard, at depths between 144–345 m during a Mareano survey in the period August 8–September 5, 2018. Funded by the Norwegian Government, the Mareano program is a collaboration between the Institute of Marine Research, the Geological Survey of Norway, and the Norwegian Hydrographic Service. Since 2006 Mareano has performed mapping of bathymetry, seafloor geology, sediment pollutants, and benthic habitats and biodiversity in Norwegian waters (see www.mareano.no). The stations were selected using a stratified random method to ensure representativity and avoid sampling bias (Buhl-Mortensen et al., 2015; Bøe et al., 2020).

Nine sediment samples for eDNA analysis (Fig. S1) were taken from five replicate faunal grab samples retrieved per station by using a two-chambered van Veen grab with a sampling area of 0.10 + 0.05 m^2^ (Norwegian Environment Agency, 2015). By using 11 cm high and 2.8 cm wide centrifuge tubes, five eDNA sediment samples were taken in each corner and in the center of the first out of five replicate faunal grab samples retrieved per station. Of these, three eDNA samples were collected from the largest and fauna-collecting chamber, covering 2% of this chamber’s surface area. In each of the remaining four grab samples, one eDNA sediment sample was taken in the center of the smallest grab chamber that was not used for fauna sampling. This strategy optimized the need for both within-grab and between-grab variations in the results from the eDNA analyses, also minimizing the outtake of sediments from the faunal samples (large chamber). All eDNA samples were taken from the top of the undisturbed grab and frozen at −80 °C.

After finishing eDNA sampling, the grab was opened and the sediment in the large chamber was sieved through 1 mm mesh. The remaining fauna was fixed in formalin/seawater and later visually identified to lowest possible taxonomic level (approximately 70% to species level). The grab was cleaned with seawater between samples. All field collections were approved by the Governor of Svalbard.

Environmental data and physical sediment parameters from each site were based on CTD and lab-standards employed in MAREANO (*e.g.*, Holte & Buhl-Mortensen, 2020).

### Fauna identification and DNA barcoding

Initial sorting and identification of fauna in the grab samples was performed by a team of taxonomists associated with MAREANO. Ongoing DNA barcoding of marine fauna collections from MAREANO and other projects contributed data for identification *via* BOLD (Boldsystems.org) (Fig. S1). Tissue samples from specimens were prepared for entry in BOLD by staff in the University Museum of Bergen (UMoB), where voucher specimens are also curated. CO1 sequencing was preformed either by the Canadian Centre for DNA Barcoding (CCDB, https://ccdb.ca) or by the DNA-lab at UMoB. Comprehensive documentation on each specimen, including sequence trace files, PCR primers, specimen photos, geographical data are available in Boldsystems.org.

### DNA extraction

To extract the DNA from the sediment we used the commercial Power Soil Pro kit (Qiagen, Hilden, Germany). From the 50 ml centrifuge tube, DNA was extracted from the sediment in two independent steps. (A) A plastic straw (0.5 cm diameter) attached to a pipette was used to subsample the top 0.5 cm of the sediment and transferred to an extraction tube. (B) Using the same type of straw, a 5 cm column was subsampled and transferred to a new tube where the sediment was homogenized and again subsampled to avoid overloading of the spin column to the extraction kit. A new clean straw was used for each subsample. After subsampling we followed the protocol for the extraction kit. Samples were extracted in a randomized way with one negative control (no sediment added) per batch of 11 samples.

### Library preparation

DNA metabarcoding was done using the Leray XT primer set, composed by mlCOIintF-XT 5′-GGWACWRGWTGRACWITITAYCCYCC-3′ (Wangensteen et al., 2018) and jgHCO2198 5′-TAIACYTCIGGRTGICCRAARAAYCA -3′ (Geller et al., 2013), targeting a 313 bp fragment of the CO1 gene. To minimize manual handling of the samples we chose to use “fusion” primers. These are primers comprising the target sequence, indexes (forward primer only), and instrument specific adaptors, resulting in a total primer length of ~60 bp. Using this approach, a second reaction (PCR, ligation) is not needed to attach indexes and adaptors to the PCR products. The metabarcoding PCRs were done in triplicate for each sample including negative controls for both extraction and PCR. PCR replicates of the same sample got the same index. The PCR amplification was verified for positive amplification and length of the fragment using a QIAxcel capillary instrument (Qiagen, Hilden, Germany). Subsequently, the samples, including negative controls, were pooled into a final library, run on an agarose gel where the correctly sized fragment was cut and purified according to protocol C of the GeneJET Gel Extraction and DNA Cleanup Micro Kit (Thermo Fisher Scientific, Waltham, MA, USA). The libraries were quantified using a Qubit dsDNA HS Kit (Thermo Fisher Scientific, Waltham, MA, USA). Further the libraries were diluted to 50 pM before loading to the Ion Chef instrument (Thermo Fisher Scientific, Waltham, MA, USA) for the final library preparation and chip loading. The libraries were sequenced on a GeneStudio S5 (Thermo Fisher Scientific, Waltham, MA, USA) using the Ion 530™ sequencing chip and the 400 bp protocol.

### Post-sequencing bioinformatics

The bioinformatic pipeline was based on the OBITools v1.01.22 software suite (Boyer et al., 2016). The removal of primer sequences was done with *ngsfilter*, allowing for two mismatches in both the forward and reverse primer sequences and none for the index sequences, before filtering the sequences on length (290–340 bp) using *obigrep*. Subsequently, the sequences were dereplicated (*obiuniq*) and chimera detection and removal performed with *vsearch* v1.10.1 (Rognes et al., 2016). Clustering of sequences into MOTUs were done with *Swarm* v2 (Mahé et al., 2015), using a *d* value of 13, which has shown to be the best trade-off between MOTU variability and the separation of intra- and inter MOTU distances (Antich et al., 2021). Singletons, that is MOTUs represented by only one sequence read were removed from the dataset before taxonomic assignment. The taxonomic assignment with *ecotag* was performed based on a locally curated reference database comprising sequences retrieved from both the EMBL database (release 117) and Barcode of Life Datasystems (BOLD) (Ratnasingham & Hebert, 2007; Wangensteen et al., 2018).

### Sequence identification and analyses of results

To validate the taxonomic assignment from the bioinformatic pipeline we used Boldigger (Buchner & Leese, 2020) with FastA-format sequences to search for matching sequences in Boldsystems.org (Ratnasingham & Hebert, 2007). Although we usually accepted the first among 20 hits from each OTU, we critically scrutinized all hits with similarities 90% or better. In most cases, the next best hits were a totally different taxon (*e.g.*, phylum) and considered an unlikely identification (similarities of 80% or less). When the next best hit was close, we consulted BOLD for more documentation. Some apparent issues were resolved because the reference material had been produced by the Natural History Museum of Bergen. Some exactly similar hits (*e.g.*, macro-algae) had different species-names. We considered such conflicts irrelevant since those taxa were not amongst our target groups. We utilized the option to produce an extra Excel sheet with the first hit for each subject and to annotate the identifications with hyperlinks to taxon pages in Gbif.org. We processed the results from the Boldigger search further by excluding sequences with less than 90% similarity. The sequences with >90% similarity from BOLD hits were additionally subjected to *blastn* search (Altschul et al., 1990) in GenBank (https://www.ncbi.nlm.nih.gov/), using a batch procedure in Geneious Prime 2021.1.1 (https://www.geneious.com) with max e-value 0.05, words-size 25, scoring match/mismatch 1 -2, and gap cost (open extend) 3 3. This allowed us to compare taxonomic annotations coming from the two databases, to assess the degree of overlap between the query and hit sequence, and to detect indels and other non-matching parts of the amplicons that were putative PCR errors. A similar procedure was performed with local *blastn* search using a downloaded sequence set of identified taxa from BOLD, acquired using the BAGS v 1.02 facility (Fontes et al., 2021). This procedure works only for publicly available sequences. Finally, we used the LULU algorithm (Frøslev et al., 2017) with a minimum ratio = 1 and minimum match at 84% settings adapted for CO1 sequences to remove OTUs assumed to have been generated from PCR and sequencing errors.

Identifications were critically validated using empirical knowledge of the Svalbard fauna (*e.g.*, https://artsdatabanken.no/), insights from ongoing DNA barcoding activities (Willassen et al., 2019) and particularly by comparison with species records from the very same samples obtained by traditional morphology-based identifications. In cases of apparent taxonomic conflicts, we sometimes adjusted questionable DNA based species identifications to higher taxonomic levels. Valid names were checked with WORMS (WoRMS Editorial Board, 2022).

Some of the identified amplicons were also aligned with their matching reference sequences to check for PCR and sequencing errors (Coissac, Riaz & Puillandre, 2012; Zhang et al., 2015; Wangensteen & Turon, 2017) and NUMTs (Lopez, Cevario & O’Brien, 1996). Additional nucleotide Blast search in NCBI (https://www.ncbi.nlm.nih.gov/) was performed in some cases to access possible intra-specific sequence variation or taxonomic discordance.

We used the web-facility http://search.norbol.org/ to examine if species identified from morphology, but were undetected with amplicons, were represented with CO1-sequences in the Bold database.

Biodiversity analyses were performed with TaxonTableTools (TTT) (Macher, Beermann & Leese, 2021), Phyloseq (McMurdie & Holmes, 2013), and Microsoft Excel®. Principal coordinates analysis (PCoA) of DNA-identified species occurrence data was performed with TTT. PCo analysis of morphologically identified taxa was done with the R package *vegan* (Oksanen et al., 2022). Both analyses were based on Jaccard distances. We used the *envfit* function in *vegan* with 999 permutations to fit environmental parameters recorded at each sampling station to the PCo ordination.

Venn-diagrams were produced at https://bioinformatics.psb.ugent.be/webtools/Venn/, and with eulerAPE (Micallef & Rodgers, 2014). Krona (Ondov, Bergman & Phillippy, 2011) was also used for Supplemental Graphics.

For the eDNA data, difference in the taxonomic composition at several levels (between fjords, among stations within fjords, surface *vs* infauna *etc*.), were done on Hellinger transformed sequence abundances by computing permutational analysis of variance (PERMANOVA) on matrices based on Bray-Curtis dissimilarities using the *adonis* function in *vegan*. Kluskal-Wallis test on occurrence data was performed in Excel.

### *A posteriori* primer testing

We assembled a set of 97 Folmer region sequences from the polychaete species identified from the samples by either morphology or DNA barcodes. The set is available in BOLD with access code DS-EDNAUMB. We used the “test with saved primers” function in Geneious Prime 2022.2.2. (https://www.geneious.com) to examine primer fit to the target sequences by allowing up to seven mismatches over the whole primer region. This could be performed only with the upstream mlCOIintF-XT, since the segment that matches primer jgHCO2198 is the terminal priming site of the Folmer segment and accordingly not part of the standard CO1 barcodes. We therefore downloaded all 319 available annelid mitochondria from GenBank (2022-08-18) to explore the match with both primers to CO1. For this test, we allowed up to six mismatches in the binding region.

## Results

### Identified taxa and amplicon abundances

A total of 14,009 and 11,137 “raw” OTUs, each with 10,593,289 and 11,028,748 reads were gained respectively from the surface and the infauna sample sets. Our search with these OTUs towards the BOLD database returned 6,712 (47.9%) and 5,374 (48.3%) matching taxonomic annotations spanning the similarity range of 50–100%. We initially excluded hits with less than 90% similarity by sorting and filtering in Excel. Several hits were BOLD sequence subjects that were previously barcoded from Mareano specimens. The result of this similarity threshold filtering is presented in Table S1 and Figs. S2 and S3. Raw data are in Tables S4 and S5.

The total number of sequence reads found in the negative controls in the surface and infauna was 323 and 345, respectively. In the surface samples the negative controls were dominated by the genus *Penicillum* (257 reads), while *Homo sapiens* dominated the infauna samples (202 reads) (Table S6). Thus, the findings in the negative controls were found not to impact the results from the eDNA samples, both because of the low number of reads and that taxa from the field samples did not appear in the negative controls. Subsequently, no further actions were needed.

Identified reads in the field samples included Proteobacteria, Amoebozoa and Ascomycota, Bacillariophyta, Haptophyta, Heterokontophyta, Ochrophyta, Pyrrophycophyta, and Rhodophyta. DNA from the photosynthetic groups, alongside with terrestrial arachnids such as *Dermatophagoides*, *Demodex*, Diptera, Lepidoptera, and intertidal *Thalassaphorura* (Collembola) were regarded as exogenous material. Some samples had DNA from fishes, *Boreogadus saida*, *Micromesistius poutassou* and from the seals *Cystophora cristata* and *Pagophilus groenlandicus*. The sediment surface layer also had DNA deposits of the semiaquatic birds, *Anser anser* and *Cepphus grylle*. We removed all these taxa from further analysis since our focus was primarily endogenous invertebrate fauna.

**Figure 2:**
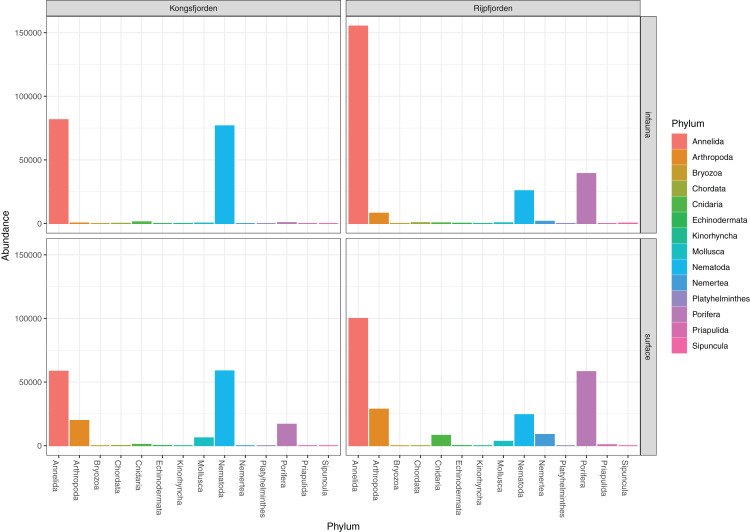
Read abundances of invertebrate phyla recovered from infauna (0–5 cm) and sediment surface (0–0.5 cm) in Kongsfjorden and Rijpfjorden.

The invertebrate sequences were a mix of probably indigenous benthic fauna and organic residues from plankton and shore habitats (Fig. 2) (Table 1). Amongst the latter were calanoid copepods, Scyphozoa and DNA from the presumably pelagic *Sarsia princeps* (Hydrozoa). Other cnidarian DNA may derive from bottom dwelling *Lucernaria* and *Plotocnide borealis*, the latter which was relatively recently found to be the medusa stage of the meiobenthic polyp known as *Boreohydra simplex* (Pyataeva et al., 2016). Because amplicons from benthopelagic organisms were sparse and because we could not determine their habitat as either benthic or pelagic, we did not exclude them from the analyses. Applying the LULU algorithm on the pooled data from the two sets of identified invertebrate reads removed 29 of 164 OTUs, including some chimeral amplicons from the nematode *Terschellingia longicaudata*. LULU also completely removed three OTUs identified with 99.6% similarity to *Scoletoma fragilis* (Polychaeta), and *Catablema vesicarium* (Hydrozoa), the latter with six reads having 100% hit.

**Table 1:** Identifications of infauna from Boldsystems (ordered by similarity to reference) and NCBI Blastn. Identified PCR and sequencing errors were assigned to corrected OTU reads with the LULU algorithm.

Bold ID	Similarity	Best match	Our ID	GenBank ID	Similarity	Best match	Bitscore	e-value	Query coverage	Max sequence	Length	Length with gaps
*Acharax* sp. CST1	90.0	GBML5547-19	*Acharax* sp.	*Acharax* sp.	89.5	LC186997	406.375	5.65E−109	97.44%	638	305	305
Amphipoda sp.	90.1	Private	Amphipoda sp.	Michthyops parvus	84.4	MK803436	273.709	4.88E−69	85.30%	395	267	267
*Acharax* sp. CST1	90.2	GBML5547-19	*Acharax* sp.	*Acharax* sp.	88.6	LC186997	367.921	2.11E−97	95.82%	633	298	299
*Acharax* sp. CST1	90.6	GBML5547-19	*Acharax* sp.	*Acharax* sp.	88.9	LC186997	367.921	1.98E−97	98.30%	624	288	289
Monostilifera sp	91.3	GBMIN138741-18	Monostilifera sp	Monostilifera sp.	91.4	KP270880	446.751	3.96E−121	100.00%	658	313	
Porifera sp.	91.8	Private	Porifera sp.	*Stephanodiscus yellowstonensis*	90.8	KT157790	427.524	2.43E−115	97.76%	582	306	306
*Acharax* sp. CST1	92.0	GBML5547-19	*Acharax* sp.	*Acharax* sp.	91.7	LC186997	440.983	2.16E−119	96.49%	636	302	302
*Serripes groenlandicus*	92.2	NBMM420-18	*Serripes groenlandicus*	*Serripes laperousii*	97.1	KF643682	514.045	2.16E−141	97.74%	420	306	306
*Eteone*	92.4	Private	*Eteone* sp.	*Eteone* sp.	84.3	MF121493	248.714	1.64E−61	86.35%	618	272	274
*Lafoea dumosa*	92.6	Private	*Lafoea dumosa*	*Lafoea dumosa*	91.3	MG935340	442.906	5.69E−120	99.36%	657	311	311
*Echinoderes svetlanae*	92.9	GBSP12389-19	*Echinoderes svetlanae*	*Echinoderes svetlanae*	93.5	NC_031873	477.514	2.17E−130	98.40%	740	308	308
*Galathowenia oculata*	93.6	NRMMC051-10	*Galathowenia oculata*	*Galathowenia oculata*	98	GU672611	446.751	3.72E−121	100.00%	660	313	
*Echinoderes svetlanae*	93.9	GBSP12389-19	*Echinoderes svetlanae*	*Echinoderes svetlanae*	93.9	NC_031873	483.282	4.00E−132	99.36%	740	311	312
*Symplectoscyphus tricuspidatus*	93.9	GBCI8872-19	*Symplectoscyphus tricuspidatus*	*Symplectoscyphus tricuspidatus*	93.9	KX095974	492.895	5.09E−135	100.00%	690	313	
*Prionospio cirrifera*	94.0	Private	*Prionospio cirrifera*	*Phalacrostemma* sp.	85.2	MN852332	108.358	2.80E−19	97.44%	602	305	305
Amphipoda sp.	94.2	Private	Amphipoda sp.	*Michthyops parvus*	83.2	MK803436	256.405	7.89E−64	85.30%	395	267	267
*Parasphaerolaimus* sp.	94.5	Early release	*Parasphaerolaimus* sp.	*Micrathena brevipes*	84.7	KJ157223	142.966	1.11E−29	43.77%	587	137	137
*Scrupocellaria* sp.	94.5	Private	*Scrupocellaria* sp.	*Tricellaria ternata*	94.2	MH243009	489.05	7.33E−134	99.36%	658	311	312
*Lucernaria janetae*	94.8	GBCI1776-13	*Lucernaria* sp.	*Lucernaria janetae*	94.6	JN700946	494.818	1.33E−135	100.00%	2781	312	
*Terschellingia longicaudata*	95.1	GBMNA5560-19	*Terschellingia longicaudata*	*Terschellingia longicaudata*	97.8	LT795770	492.895	4.98E−135	89.25%	621	274	274
*Haploposthia rubra*	95.3	GBSP4371-12	*Haploposthia rubra*	*Haploposthia rubra*	97.8	FR837862	562.112	7.41E−156	100.00%	657	313	
*Laphania boecki*	95.7	CCANN770-09	*Laphania boecki*	Terebellidae sp.	95.7	HM375494	473.669	3.12E−129	90.10%	629	282	282
*Aphelochaeta*	95.8	Private	*Aphelochaeta*	*Aphelochaeta* sp.	79.9	MK971219	239.101	1.28E−58	100.00%	657	313	
*Plicatellopsis fragilis*	95.9	GBMIN44292-15	Plicatellopsis bowerbanki	*Phakellia bowerbanki*	97.2	MK561021	496.741	3.40E−136	93.38%	628	282	282
*Yoldiella frigida*	96.2	ABMBS194-10	*Yoldiella frigida*	*Yoldiella frigida*	100	HQ919186	602.489	5.19E−168	100.00%	658	313	
*Galathowenia oculata*	96.3	CHONE215-11	*Galathowenia oculata*	*Galathowenia oculata*	95.4	GU672578	414.065	2.83E−111	86.15%	600	253	255
*Terschellingia longicaudata*	96.3	GBMNA5560-19	*Terschellingia longicaudata*	*Terschellingia longicaudata*	97.5	LT795770	502.509	6.97E−138	84.43%	597	282	282
*Ciliatocardium ciliatum*	96.6	ARMOL028-12	*Clinocardium ciliatum*	*Clinocardium ciliatum*	99.7	KF644063	590.953	1.55E−164	100.00%	658	313	314
*Adontorhina*	97.0	NBMM754-19	*Adontorhina*	*Mesochorus* sp.	87.4	HQ927238	104.513	4.39E−18	26.61%	440	87	87
*Terschellingia longicaudata*	97.1	GBMNA5560-19	*Terschellingia longicaudata*	*Terschellingia longicaudata*	96.7	LT795770	415.988	7.31E−112	76.97%	557	243	244
*Terschellingia longicaudata*	97.4	GBMNA5560-19	*Terschellingia longicaudata*	*Terschellingia longicaudata*	97.4	LT795770	544.808	1.17E−150	100.00%	621	307	
*Terschellingia longicaudata*	97.4	GBMNA5560-19	*Terschellingia longicaudata*	*Terschellingia longicaudata*	97.4	LT795770	544.808	1.28E−150	92.47%	621	307	307
*Terschellingia longicaudata*	97.4	GBMNA5560-19	*Terschellingia longicaudata*	*Terschellingia longicaudata*	91	LT795770	289.091	1.09E−73	100.00%	593	279	299
*Terschellingia longicaudata*	97.4	GBMNA5560-19	*Terschellingia longicaudata*	*Terschellingia longicaudata*	95.4	LT795770	423.679	3.48E−114	89.74%	588	274	280
*Galathowenia oculata*	97.5	CHONE215-11	*Galathowenia oculata*	*Galathowenia oculata*	98	GU672611	456.364	5.05E−124	93.50%	638	291	303
*Terschellingia longicaudata*	97.7	GBMNA5560-19	*Terschellingia longicaudata*	*Terschellingia longicaudata*	97.7	LT795770	475.591	8.92E−130	78.64%	579	265	265
*Terschellingia longicaudata*	97.9	GBMNA5560-19	*Terschellingia longicaudata*	*Terschellingia longicaudata*	97.1	LT795770	414.065	2.98E−111	71.68%	618	243	244
*Cossura longocirrata*	97.9	Private	*Cossura longocirrata*	*Tanytarsus thomasi*	78.3	JN265092	179.497	1.05E−40	93.56%	641	277	277
*Cossura longocirrata*	98.0	Private	*Cossura longocirrata*	*Tanytarsus thomasi*	78.4	JN265092	194.879	2.62E−45	91.69%	651	287	287
*Nicomache minor*	98.1	GBAN18134-19	*Nicomache minor*	*Nicomache minor*	97.8	MG975588	546.731	3.14E−151	100.00%	669	313	
*Terschellingia longicaudata*	98.1	GBMNA5560-19	*Terschellingia longicaudata*	*Terschellingia longicaudata*	96.7	LT795770	348.694	1.29E−91	68.29%	598	223	224
*Terschellingia longicaudata*	98.1	GBMNA5560-19	*Terschellingia longicaudata*	*Terschellingia longicaudata*	97.8	LT795770	394.838	1.76E−105	68.71%	587	212	214
*Leitoscoloplos pugettensis*	98.4	BEST012-08	*Leitoscoloplos pugettensis*	*Leitoscoloplos pugettensis*	99	HM473770	573.648	2.47E−159	91.99%	658	313	313
*Lafoea dumosa*	99.0	Private	*Lafoea dumosa*	Lafoeidae sp.	94.6	MG422454	504.432	1.71E−138	100.00%	658	313	
*Cephalothrix iwatai*	99.0	GBSP10430-18	*Cephalothrix iwatai*	*Cephalothrix iwatai*	99	KP270873	585.185	8.40E−163	100.00%	658	313	
Gymnolaemata	99.2	Private	Gymnolaemata	Lumbricidae sp.	78.8	GU014092	187.188	5.91E−43	81.71%	654	278	278
*Aphelochaeta* sp.	99.4	Private	*Aphelochaeta* sp.	*Aphelochaeta* sp.	80.4	MK971172	231.41	2.64E−56	99.68%	666	311	311
*Gyptis golikovi*	99.4	Private	*Gyptis golikovi*	*Gyptis mackiei*	98.2	DQ442562	494.818	1.34E−135	86.86%	571	271	271
*Boltenia echinata*	99.4	WSVAR010-09	*Boltenia echinata*	*Boltenia echinata*	98.4	MG422647	567.88	1.36E−157	100.00%	509	313	314
*Aricidea quadrilobata*	99.5	Early release	*Aricidea quadrilobata*	*Aricidea* sp.	81.4	KY805818	242.946	8.82E−60	94.53%	653	295	295
*Acartia longiremis*	99.5	ECHAR407-19	*Acartia longiremis*	Uncultured marine	98.1	KT186357	542.885	4.58E−150	100.00%	657	313	316
*Maldane sarsi*	99.6	Early release	*Maldane sarsi*	*Maldane sarsi* CMC01	97.9	HQ023885	500.586	2.30E−137	100.00%	660	313	
*Scoletoma fragilis*	99.6	Private	*Scoletoma fragilis*	*Lumbrineris* sp.	96.6	HM473776	500.586	2.58E−137	88.69%	635	290	290
*Ophiacantha bidentata*	99.6	Private	*Ophiacantha bidentata*	*Ophiacantha bidentata*	99	KU495769	558.267	1.07E−154	98.41%	654	309	311
*Halcampa decemtentaculata*	99.6	Private	*Halcampa* sp.	*Halcampa chrysanthellum*	98.7	MG935150	564.035	2.04E−156	95.40%	658	313	313
*Halcampa decemtentaculata*	99.6	Private	*Halcampa* sp.	*Halcampa chrysanthellum*	99	MG935150	575.571	6.54E−160	100.00%	658	312	
*Plotocnide borealis*	99.6	GBCI8722-19	*Plotocnide borealis*	*Plotocnide borealis*	99	KU721808	564.035	1.95E−156	100.00%	654	313	314
*Ascidia callosa*	99.7	Private	*Ascidia callosa*	*Ascidia virginea*	73.9	MW363030	117.971	3.69E−22	90.03%	626	280	280
Gymnolaemata	99.7	Private	Gymnolaemata	Japygidae sp.	79.2	MT902579	219.874	7.83E−53	96.81%	658	303	303
*Molpadia borealis*	99.7	Private	*Molpadia borealis*	*Molpadia musculus*	84.6	HM196468	323.699	4.36E−84	99.68%	469	312	312
*Eteone* cf. *flava*	99.7	Private	*Eteone* sp.	*Eteone longa*	85.7	HM417789	344.849	2.03E−90	93.18%	662	314	314
*Eteone* cf. *flava*	99.7	Private	*Eteone* sp.	*Eteone flava*	85.8	KR916815	342.926	7.11E−90	99.04%	657	310	310
*Plawenia*	99.7	ALPNB050-14	*Plawenia*	*Siboglinum fiordicum*	87.5	KJ789170	356.385	6.31E−94	94.57%	13112	296	296
*Chaetozone*	99.7	BBPS319-19	*Chaetozone* sp.	*Tharyx* sp.	99.4	HQ023817	590.953	1.54E−164	100.00%	620	313	
*Pseudocalanus acuspes*	99.7	Early release	*Pseudocalanus acuspes*	*Pseudocalanus* sp.	99.4	HQ966475	590.953	1.54E−164	99.68%	657	312	312
*Sarsia princeps*	99.7	CNNN068-08	*Sarsia princeps*	*Sarsia princeps*	99.7	MG421639	596.721	2.83E−166	100.00%	658	313	
*Scoletoma fragilis*	99.7	CCANN577-09	*Scoletoma fragilis*	*Scoletoma fragilis*	99.7	MG422040	596.721	2.83E−166	100.00%	658	313	
Spionidae	99.7	CCPOL337-08	Spionidae	Spionidae sp.	99.7	HQ024464	596.721	2.83E−166	100.00%	660	313	
*Aphelochaeta* sp.	100.0	sp EO	*Aphelochaeta* sp.	*Kirkegaardia* sp.	79.7	KY775641	235.255	1.83E−57	92.97%	605	291	291
*Cossura pygodactylata*	100.0	Private	*Cossura pygodactylata*	*Cladopelma galeator*	80.2	JF871410	217.951	2.96E−52	89.10%	652	278	278
*Prionospio cirrifera*	100.0	Private	*Prionospio cirrifera*	*Prionospio* sp.	80.3	KT307691	241.023	3.37E−59	35.76%	446	107	108
*Cossura pygodactylata*	100.0	Private	*Cossura pygodactylata*	*Diplocirrus toyoshioae*	80.4	LC314567	223.719	5.47E−54	89.49%	675	281	281
*Microcalanus pusillus*	100.0	CAISN325-12	*Microcalanus pusillus*	Arthropoda sp.	80.4	MN690131	242.946	8.88E−60	97.76%	313	306	306
*Abyssoninoe* sp.	100.0	Private	*Abyssoninoe* sp.	*Melinnopsis gardelli*	80.5	MT556177	229.487	1.00E−55	91.69%	632	287	287
*Golfingia margaritacea*	100.0	Early release	*Golfingia margaritacea*	*Phascolopsis gouldii*	80.8	DQ300134	256.405	7.89E−64	100.00%	654	313	
*Eupraxillella* sp.	100.0	Early release	*Eupraxillella* sp.	Euclymeninae sp.	81.3	LC342659	254.482	3.00E−63	98.73%	658	309	310
*Lumbriclymene* sp.	100.0	Early-Release	*Lumbriclymene* sp.	*Stomatia obscura*	81.6	AB505294	235.255	1.82E−57	87.46%	638	272	272
*Chaetozone setosa*	100.0	Priv WSBP1298-15	*Chaetozone* WS	*Kirkegaardia* sp.	81.9	KY775641	254.482	2.99E−63	92.01%	666	288	288
*Chaetozone setosa*	100.0	Priv WSBP1306-15	*Chaetozone* WS	*Kirkegaardia* sp.	82	KY775641	262.173	1.57E−65	87.28%	673	295	295
*Chaetozone setosa*	100.0	Priv WSBP1297-15	*Chaetozone* WS	*Kirkegaardia* sp.	82.2	KY775641	194.879	2.43E−45	77.05%	602	224	225
*Pleusymtes glaber*	100.0	Early-Release	*Pleusymtes glaber*	Chiltoniidae sp.	82.9	KT958229	250.637	4.30E−62	87.86%	355	274	275
*Microclymene* sp.	100.0	Private	*Microclymene* sp.	*Clymenella collaris*	84.6	LC342660	323.699	4.35E−84	100.00%	658	312	
Sabellidae	100.0	Private	Sabellidae	Sabellidae sp.	92.3	HM473799	460.21	3.51E−125	99.36%	657	311	311
*Heteromastus filiformis*	100.0	Early release	*Heteromastus filiformis*	*Barantolla americana*	95.8	HM473729	527.504	1.94E−145	100.00%	654	313	
*Iophon* sp. 1 PRT-2020	100.0	GBMNC37348-20	*Iophon* sp. 1 PRT-2020	*Iophon* sp.	98.4	MT491721	525.581	7.36E−145	91.05%	641	285	285
*Scoloplos* sp.	100.0	Early release	*Scoloplos* sp.	*Scoloplos* sp.	98.4	GU672377	565.958	5.10E−157	99.68%	656	309	309
*Leitoscoloplos pugettensis*	100.0	BEST012-08	*Leitoscoloplos pugettensis*	*Leitoscoloplos pugettensis*	99	HM473770	573.648	2.71E−159	100.00%	658	313	
*Pseudocalanus acuspes*	100.0	Early release	*Pseudocalanus acuspes*	*Pseudocalanus moultoni*	99.4	JX995281	589.03	5.85E−164	100.00%	635	313	
*Chaetozone* sp.	100.0	GBMNC20421-20	*Chaetozone setosa*	*Chaetozone* sp.	99.7	MT065931	590.953	1.55E−164	100.00%	658	313	314
*Halocynthia pyriformis*	100.0	CCSMA042-07	*Halocynthia pyriformis*	*Halocynthia pyriformis*	99.7	FJ528610	596.721	2.83E−166	100.00%	673	313	
*Maldane sarsi* CMC02	100.0	NUNAV-0092	*Maldane sarsi*	*Maldane sarsi*	99.7	HQ023885	590.953	1.55E−164	95.58%	630	282	282
*Nicomache lumbricalis*	100.0	GBAN18133-19	*Nicomache lumbricalis*	*Nicomache lumbricalis*	99.7	MG975595	590.953	1.55E−164	100.00%	663	313	314
*Polycirrus arcticus*	100.0	Early release	*Polycirrus arcticus*	*Polycirrus arcticus*	99.7	MT167015	596.721	2.83E−166	100.00%	681	313	
*Yoldiella nana*	100.0	Private	*Yoldiella nana*	*Yoldiella nana*	99.7	HQ919200	590.953	1.55E−164	100.00%	658	313	314
*Balanus balanus*	100.0	Private	*Balanus balanus*	Balanus sp.	100	MG317264	602.489	5.19E−168	100.00%	658	313	
*Calanus hyperboreus*	100.0	CAISN1094-13	*Calanus hyperboreus*	Calanus sp.	100	KF931007	602.489	5.19E−168	100.00%	658	313	
*Ctenodiscus crispatus*	100.0	CHONE008-10	*Ctenodiscus crispatus*	*Ctenodiscus crispatus*	100	HM405877	602.489	5.19E−168	100.00%	658	313	
*Cyanea* sp.	100.0	GBCI11193-19	*Cyanea* sp.	*Cyanea capillata*	100	MG423436	602.489	5.19E−168	100.00%	658	313	
*Cyanea* sp.	100.0	GBCI11196-19	*Cyanea* sp.	*Cyanea* sp.	100	MG421562	602.489	5.19E−168	100.00%	658	313	
*Dodecaceria concharum*	100.0	GBAN0672-06	*Dodecaceria concharum*	*Dodecaceria concharum*	100	KP794934	602.489	5.19E−168	100.00%	658	313	
*Galathowenia oculata*	100.0	WSPO073-09	*Galathowenia oculata*	*Galathowenia oculata*	100	GU672611	602.489	5.19E−168	80.51%	599	252	252
*Laonice cirrata*	100.0	GBAN18009-19	*Laonice cirrata*	*Laonice cirrata*	100	MG234459	598.643	7.41E−167	100.00%	629	311	
*Lumbrineris mixochaeta*	100.0	BEST018-08	*Lumbrineris mixochaeta*	*Lumbrineris* sp.	100	HM473776	602.489	5.19E−168	100.00%	658	313	
*Maldane sarsi*	100.0		*Maldane sarsi*	*Maldane sarsi*	100	GU672596	602.489	5.19E−168	100.00%	658	313	314
*Pholoe assimilis*	100.0	GBMNB35948-20	*Pholoe assimilis*	*Pholoe baltica*	100	GU672501	602.489	5.19E−168	100.00%	660	313	
*Strongylocentrotus droebachiensis*	100.0	Private	*Strongylocentrotus droebachiensis*	*Strongylocentrotus droebachiensis*	100	LN828961	606.334	3.74E−169	97.52%	615	315	315
*Strongylocentrotus pallidus*	100.0	GBEH1822-08	*Strongylocentrotus pallidus*	*Strongylocentrotus pallidus*	100	KF643004	602.489	5.19E−168	100.00%	658	313	

### Comparing BOLD-ID hits with GenBank hits

Blastn search with our similarity filtered invertebrate sequences presented several conflicting best hits, many of which had lower similarity values in the GenBank hits. This indicated poorer taxon coverage in the GenBank database (Table 1), and that many of the hits in BOLD were still not represented in GenBank. For example, the best hits in GenBank for some polychaete sequences were Diptera with about 80% similarity. Other conflicts indicated that GenBank is not always a reliable source for taxonomic annotation (*e.g.*, see Locatelli et al. (2020) reply to Leray et al. (2019)). Some of the species level conflicts may also be ascribed to misidentifications of BOLD vouchers. Taxonomic “bin discordance” in BOLD may represent either errors or poorly understood species (Radulovici et al., 2021). One advantage with GenBank over BOLD is the report of sequence coverage and alignment gaps. Blastn may reveal PCR-errors and chimeric sequences that show high similarity over a short segment of the target sequence. Such high similarity hits may contribute to inflation of OTU numbers (Dickie, 2010; Coissac, Riaz & Puillandre, 2012). We generally accepted the annotations from Bold that had higher similarities than those in GenBank. However, in some cases we also revised the OTU-annotation from BOLD (Table 1). (See “Discussion” and Text S1 for comments on annotations).

### Curated identifications

**Figure 3:**
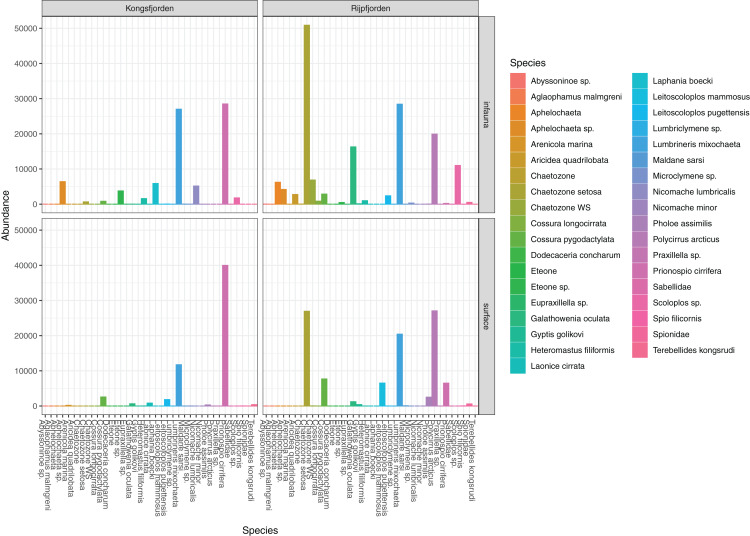
Read abundances of infauna (0–5 cm) and sediment surface (0–0.5 cm) polychaete taxa from Kongsfjorden and Rijpfjorden.

The genuine benthic fauna (as opposed to pelagic and other exogenous animals) was dominated by polychaetes and nematodes (Fig. 2). *Lumbrineris mixochaeta*, *Prionospio cirrifera, Chaetozone* spp., *Galatowenia oculata*, *Polycirrus arcticus* and *Scoloplos* sp. were some of the more abundant of the 38 polychaete taxa (Fig. 3). We recorded both *Cossura pygodactylata* and *Cossura longocirrata*, two species that have sometimes been confused (Zhadan, Vortsepneva & Tzetlin, 2012).

Also relatively abundant, but much less species rich, were the nematodes. Our collection of *Terschellingia longicaudata* amplicons diverged about 10% and matched two reference library bins, BOLD:ADR5938 and BOLD:ADR5935. The best hit in GenBank was accession number LT795770.

**Figure 4:**
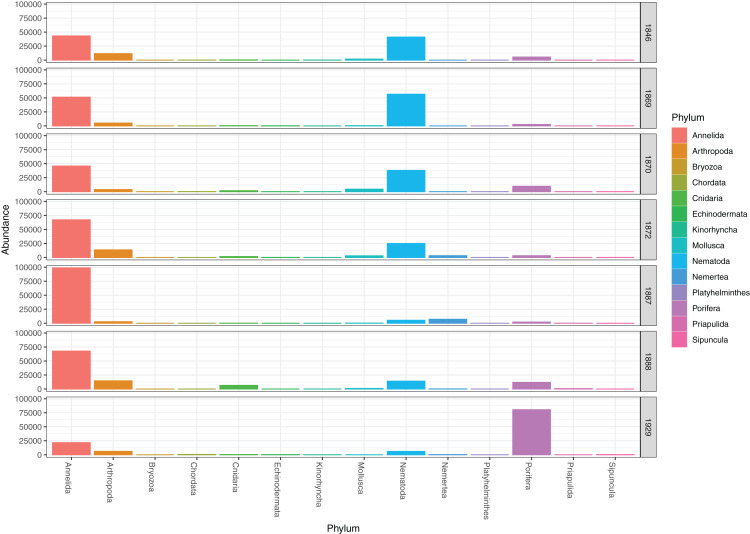
Pooled read abundances of invertebrate phyla from the sampling stations in Kongsfjorden (R1846–1870; see Fig. 1) and Rijpfjorden (R1872–1929).

DNA from other invertebrate phyla was relatively sparse (Fig. 2) and unevenly dispersed over the samples (Fig. 4), reflecting patchy and possibly clumped distributions. We recorded three species of Ascidiacea, two Bryozoa, seven Echinodermata, seven Mollusca, one Turbellaria, one Priapulida, and one Sipuncula. The Arthropoda, Cnidaria, Echinodermata, Mollusca and Porifera were slightly more abundant in sediment surface (Table S2) than in the homogenized deeper sediments (infauna, Table S3) (Fig. 2). Porifera were also more abundant in Rijpfjord than in Kongsfjord (Fig. 2).

### Alpha diversities

**Figure 5:**
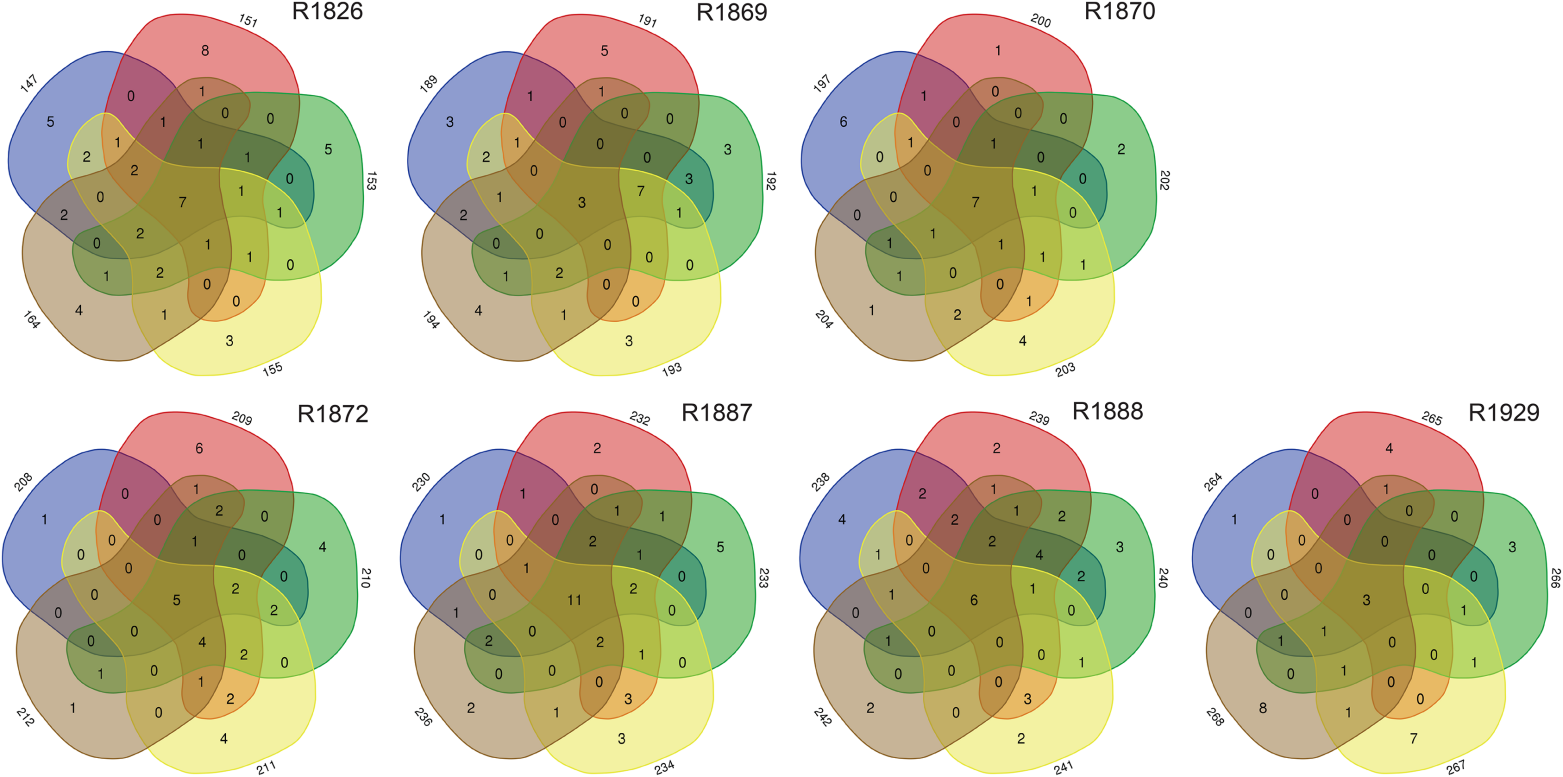
Numbers of unique and shared morphologically identified species of polychaetes in grab replicates (three digits peripheral sample codes) from the seven sampling stations in Kongsfjord (R1846–1870) and Rijpfjord (R1872–1929).

Examination of morphologically identified polychaetes in grab replicates showed that relatively few species, 7–25% (mean 14.3, std 6.3), were shared by all five grab replicate samples from a station. Totally 30–68% (mean 42.5, std 13.1) of the species recorded from a sampling station were observed in just one of the five grab replicates (Fig. 5).

**Figure 6:**
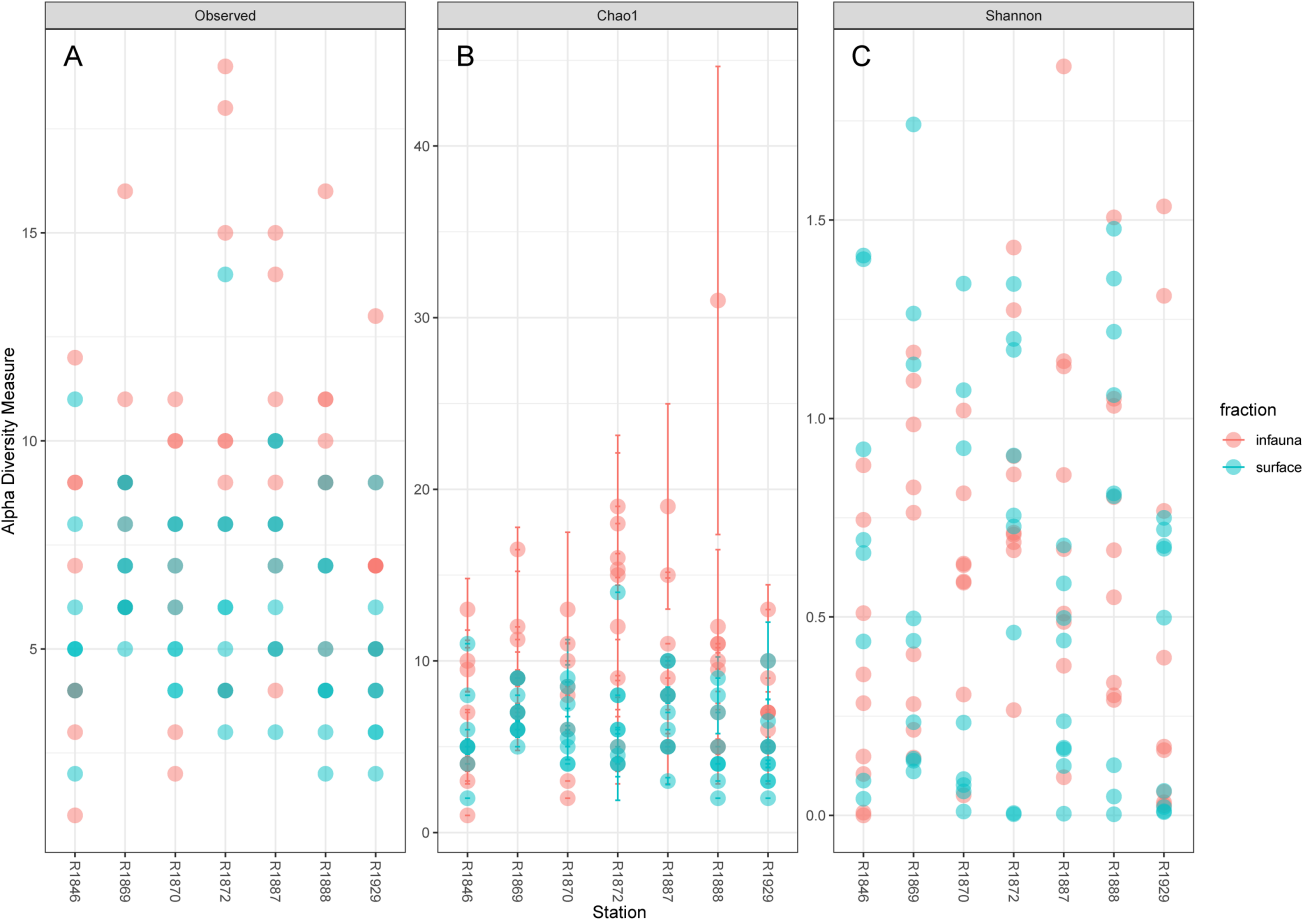
Plot of alpha diversity in metabarcoded samples. Blue dots, surface samples; red dots, grab samples; mixed colors, superimposed data points. (A) Species richness; (B) Chao index with estimates of undetected taxa; (C) Shannon’s diversity index.

Considerable variability in numbers of invertebrate taxa detected with eDNA from replicate grab samples at each station was also observed (Table 2, Fig. 6), probably in part reflecting the heterogeneous spatial distribution of the animals also observed visually (Fig. 5). The Chao1 index aims to model undetected taxa, based on observations and abundances of especially the rare taxa. Our estimates of Chao1 had standard errors (Fig. 6) from 0 to maximum 13.64, in the latter case with exceptional estimates of 15 infauna species more than observed in one of the replicates from station R1881(Fig. 6). However, especially low richness counts usually had the same estimates with Chao1. Shannon’s index showed several examples of values near zero (Fig. 6), representing 1–3 species in one sample and with similar tendencies to those seen in morphology-based taxa counts (Fig. 5).

**Table 2:** Numbers of DNA-detected invertebrate species pooled from all five grabs *vs* the number pooled from five intra grab replicates from one grab.

	Kongsfjord			Rijpfjord			
	R1846	R1869	R1970	R1972	R1887	R1888	R1929
Tot spp at station	24	26	29	39	36	32	26
Tot spp in intra grab replicates	18	23	22	38	36	29	18

The pooled reads from the intra grab replicates rarely covered the full variability span of species numbers from the four other grabs (Table 1), except for grab surface sample 236 and infauna sample 212 from Rijpfjord. This indicates that intra grab replicates may increase the number of detections, but that grab replicates also need to account for the uneven spatial distribution of the taxa, as expressed also in the eDNA data by Shannon’s diversity index (Fig. 6).

**Figure 7:**
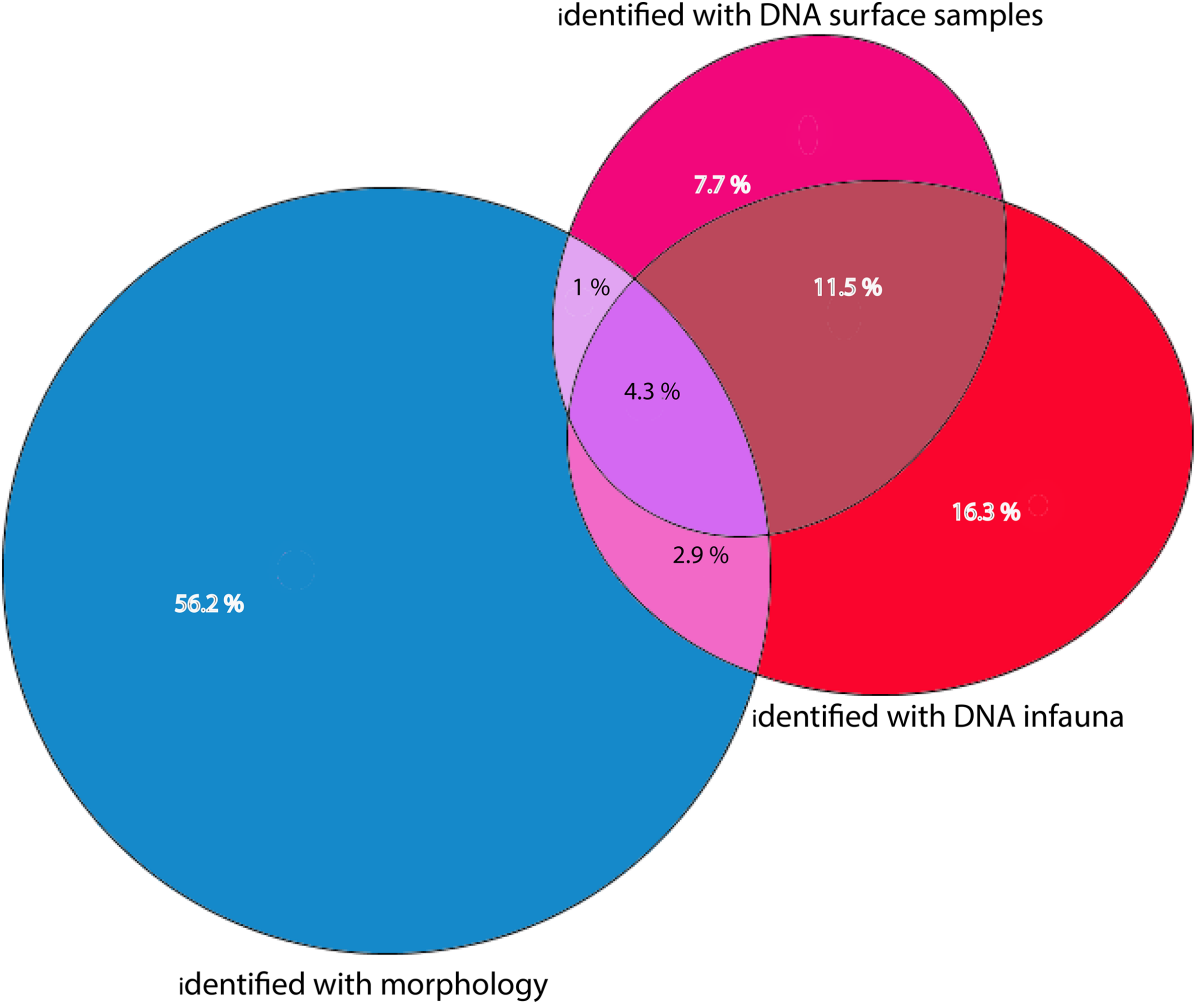
Percentages of total numbers of invertebrate taxa identified from all sampling stations combined. Morphology identified sums to 64.4% of totals, eDNA surface samples 24.5%, and eDNA infauna samples 35%. Proportions of shared taxa sets in overlapping sections.

Composite data from infauna, and surface respectively, usually measured more taxa in the former sets (Figs. 6 and 7), a result that could be due to real diversity differences between two habitat types, but also an additional effect of different sample volumes.

### Morphological *vs* DNA-based identifications

Morphologically identified taxa were 64.4% of the total numbers of taxa recorded from the three sets of occurrence data. DNA-identified surface samples represented 24.5% of the taxa and infauna 35% (Fig. 7). Proportions of overlapping sets revealed only 8.2% of shared taxa between morphology identified and DNA-identified taxa. Kluskal-Wallis testing (H = 16.58, *p* = 3.4-08, alfa = 0.01) revealed significant differences in numbers of species in the three sets of data. A considerable proportion of the morpho-species were not detected with DNA (Fig. 7). When comparing species numbers from the two approaches, one should bear in mind that many species detections from eDNA are animals that were not targeted in the morphological (visual) sorting and identification work. We counted 74 taxa with DNA that were not reported from morphological identifications. The detection of a few species that are routinely not identified to species level in benthic surveys, such as kinorhynchs, nematodes, nemertea, and hydroids, testifies to one of the advantages of a DNA-based approach, if the aim is to increase detection of less conspicuous species.

**Figure 8:**
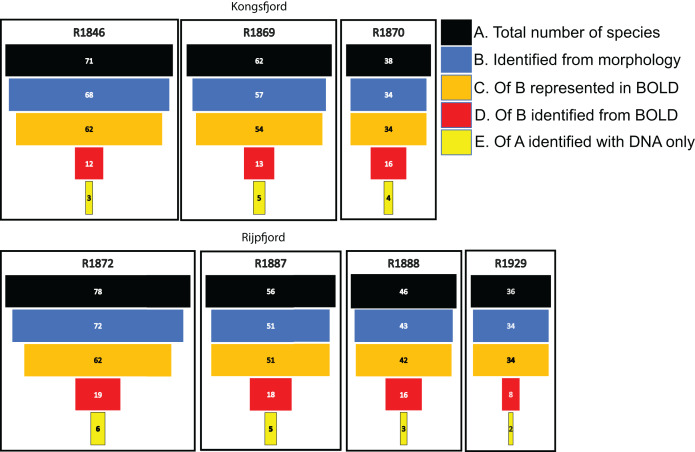
Species numbers of polychaetes identified with different approaches in this study. Notice the number of species identified with morphology that were not detected with eDNA, despite representation with barcode sequences in http://boldsystems.org/.

For polychaetes, DNA-identification also detected 2–6 species at each station that were not observed with visual identification (Fig. 8). However, only 17% to 47% (mean 20.6%) of the polychaete species identified with morphology were also detected with DNA. For instance, at station R1846 only 12 polychaete taxa were detected with eDNA whilst morphology identified 68. We observed that 62 of those 68 taxa are represented with barcodes in BOLD. Searching BOLD with taxa names indicated that 90–100% (mean 96.7%) of the polychaete species identified from morphology were also represented with at least one sequence in BOLD (Fig. 8). This suggests that other factors than lack of barcodes in BOLD (Kvist, 2013; Weigand et al., 2019; Hestetun et al., 2020; Mugnai et al., 2021) are responsible for the missing detections.

### Primer mismatch

Over the set of polychaete sequences, we observed up to seven 5′ Watson-Crick (W-C) mlCOIintF-XT primer mismatches in a sequence. Several of these were sites where a degeneracy of two was insufficient for W-C pairing. The mismatches were in positions 1, 2, 3, 4, 6, 9, 15,19, 24 of the 26 bases long oligonucleotide. Inosine pairings were not counted as mismatches. The pairings were either G:G, A:G, C:C, C:T, T:C, A:C, T:G, forming more or less weaker pairings than ordinary Watson-Crick primer-template bindings. Many of the undetected species had one or more primer mismatch(es) (Fig. S4). However, several of the polychaete species with no mismatches in the forward primer were not detected, although fair numbers of individuals were recorded with visual sorting of the grab (Fig. S5). At the 3′ end, which is considered most important for the priming efficiency, Geneious found a mismatch in 27 sequences in degenerate position 24. A somewhat similar picture of mlCOIintF-XT mismatch was expressed in the data from 319 annelid mitochondria, indicating some particular mismatches with Clitellata and Myzostomida. These data gave the impression of an overall better match with primer jgHCO2198, showing usually 0–2 mismatches only, and none in the 3′ region.

### Community analysis

**Figure 9:**
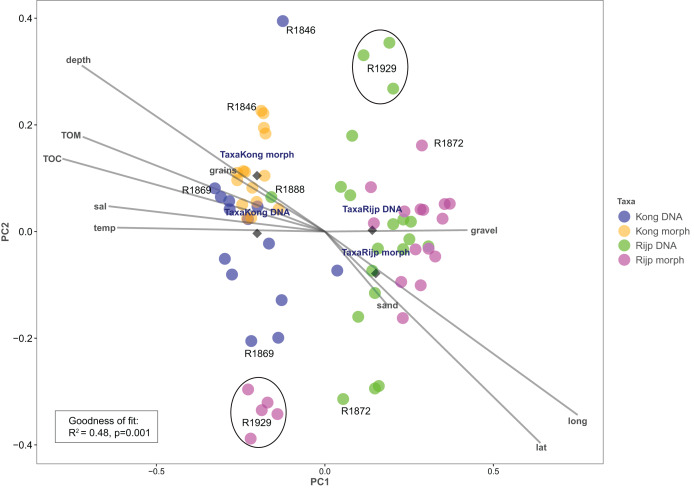
Plot of principal coordinates analysis (PCoA) of eDNA data (DNA) (presence-absence) and morphologically identified taxa (morph) (presence-absence) from seven sampling stations in Kongsfjord (Kong) and Rijpfjord (Rijp). Diamonds = centroids. Select samples are marked with station codes (see Fig. 1). Fitted environmental variables: latitude (lat), longitude (long), bottom depth (depth), temperature (temp), and salinity (sal), percent total organic matter (TOM), percent total organic carbon (TOC), percent sand (sand), percent gravel (gravel), and percent grain size (grains).

Principal coordinates analysis based on eDNA absence-presence data returned ANOSIM R = 0.43 with *p* < 0.001. The first and second axes explained 15.6% and 10.9% of the variation. PCoA of the morphologically identified occurrence data returned respectively 21.5% and 9.5% explained on the first and second axes. Both analyses displayed some separation of the samples from the two fjords on the first axis (Fig. 9). The second axis showed the eDNA samples slightly more dispersed than the morphology-based samples. The five morphology-identified samples from station R1929 particularly separated from the remaining samples on both axes. We fitted the following variables to the ordination: latitude (lat), longitude (long), bottom depth (depth), temperature (temp), and salinity (sal), percent total organic matter (TOM), percent total organic carbon (TOC), percent sand (sand), percent gravel (gravel), and percent grain size (grains) (Fig. 9, Table S7). We found all these variables, except percent gravel, as significant vectors, with geography and depth particularly contributing to the total goodness of fit of R^2^ = 0.45 of the eDNA data and R^2^ = 0.50 of the morpho-data. Organic matter and total organic carbon were also highly significant and appear to explain a considerable part of the difference in faunal composition between the two fiords, particularly as estimated from the morpho-data (Table S7).

The PERMANOVA tests, based on read abundances, also revealed a significant difference in taxon composition between fjords, both overall and when subdividing the data into surface and infauna (*p* < 0.001, Table S8). Comparisons among stations within the two fjords revealed a significant difference in Rijpfjorden (*p* < 0.001), but not in Kongsfjorden (*p* = 0.63). In both fjords a significant difference was found between surface and infauna, which derives from the detection of more taxa in the infauna samples.

## Discussion

### Separating signal from noise

Metabarcoding has increasingly been approved as an alternative approach to explore and assess species diversity in ecosystems. It has also been advocated as a method that may supersede traditional discovery and monitoring of rare species due to the sensitivity of the PCR method (*e.g.*, Holman et al., 2019). Our results indeed are complementing a traditional approach because we found species that are not identified in standard surveys, such as nematodes, hydrozoans, porifera and kinorhynchs (but see Bijoy Nandan et al., 2016; Somerfield et al., 2006). We also detected species that are likely to be confused with close congeners and have not been documented in Svalbard waters before, such as *Leitoscoloplos pugettensis* and *Cossura pygodactylata*. However, totally 95 of the benthic species recorded with traditional sorting and identification were not detected with eDNA. Of the 74 species that were recorded with eDNA only, several may trace to pelagic or other habitats, thus contributing to less taxa overlap between the two approaches (Fig. 7). Considerable influx of suspended material from the photic or littoral zone, as well as from terrestrial sources was additionally demonstrated by eDNA identifications. Some of the eDNA may also have been redistributed by activities of mobile animals. Such transported DNA may disturb and complicate interpretation of ecological conditions in the study habitat. However, if one can recognize the original source of such signals, they may contribute interesting point observations to a broader understanding of local aspects of a land-ocean aquatic continuum (Xenopoulos et al., 2017) and ecological processes in the environment (Rees et al., 2014; Roussel et al., 2015). Because we used a taxon-based approach, we were able to categorize such OTUs as non-target taxa.

Our somewhat noisy data, with respect to environmental origin, reflect the dilemma that multispecies surveys depend on degenerate primers that will also amplify non-target sequences (Brandon-Mong et al., 2015; Zinger et al., 2019). The Leray primer set (Leray et al., 2013) was designed to cover all metazoan diversity. We used the pair called Leray-XT, which has also been used to target hard-bottom fauna (Wangensteen et al., 2018) and presumed to work well on marine invertebrates. In line with common procedure, we pooled triplicate PCR reactions, with the intention to reduce bias due to random effects and chimera formation (Marotz et al., 2019). However, competing non-target DNA may prevent detection of targeted species so that high proportions of the fauna fail detection with metabarcoding (Alberdi et al., 2018; Collins et al., 2019; Cowart et al., 2015; Elbrecht & Leese, 2015). Biased PCR efficiency occurs due to the thermodynamics of primer pairing, causing different primer variants to bind to different templates with different strengths at the annealing step of the PCR reaction. Experiments with the bacterial 16S have shown that PCR selection, resulting in overamplification of more reactive templates, may be caused by differences in the GC content at degenerate positions in the primer target sites (Polz & Cavanaugh, 1998). This bias may also misrepresent the amounts of sequence reads (Stadhouders et al., 2010), if taken as proxies of specimen abundance or biomass. Additional confounding effects are created from PCR-errors such as single nucleotide indels and longer inserts or deletions resulting from self-priming or hybridizing strands (Gołezbiewski & Tretyn, 2019; Zhang et al., 2015). We discovered examples of such chimeric OTUs from amplified *Terschellingia longicaudata* sequences.

PCR-generated problems may have stronger bearings on biodiversity studies that rely on quantified species metrics, but PCR may certainly also affect presence-absence records and the detection of particular species that tend to exhibit some degree of inertia to standardized PCR-protocols (Dell’Anno et al., 2015; Elbrecht & Leese, 2015; Ficetola et al., 2008; Wilcox et al., 2013). Our own work with barcoding invertebrates has shown that “universal primers” (with various modifications) may fail to amplify the Folmer region of CO1 of several taxa and this explains some of the missing taxa in Boldsystems (Weigand et al., 2019). However, here we failed to detect many of the taxa that are represented in the barcode reference library (Fig. 8), and it seems plausible that PCR bias is one reason for the weak correspondence between DNA based and morphology-based identifications (Figs. S4 and S5) (Cowart et al., 2015). Insights from real time qPCR (Stadhouders et al., 2010) suggest that primer-template association and dissociation kinetics will have considerable differentiating effects on the amplification of the variety of potential template DNAs in an environmental sample. In the set of polychaete sequences, we observed several deviations from ordinary Watson-Crick base-pairing, such as A:G, C:C, C:T, T:C, A:C, T:G, where particularly the first two types of mismatches may have noticeable priming effects (Stadhouders et al., 2010; Simsek & Adnan, 2000). We also observed that the inosine (I) nucleotide in the primer was often associated with template guanine (G), which is a weak bond, as compared with the other DNA nucleotides (Ben-Dov & Kushmaro, 2015). It is, however, difficult to assess how the kinetics of primer-template hybridization will determine the outcome of sequence reads in such diverse mixed samples of eDNA.

Analyses of eDNA rest on detection of either exact sequence variants (ESVs, also called ZOTUs, ASVs, centroids) or the construction of operational taxonomic units (OTUs) (*e.g.*, Glassman & Martiny, 2018; Porter & Hajibabaei, 2020; Pappalardo et al., 2021). The OTU-based approach, applied in this study, can be used to compute “taxon-free” biodiversity statistics with the intension to understand ecological differences between samples (*e.g.*, Mächler, Walser & Altermatt, 2020). Alternatively, the sequence reads can be regarded as “closed reference OTUs” if they are sufficiently similar to labelled sequences in a reference database, However, because *de novo* OTUs are shaped by pairwise comparison with other data in the set, they are context dependent and difficult to compare with OTUs from other datasets (Callahan, McMurdie & Holmes, 2017). Also, if OTUs cannot be referred to biological species that are linked with a minimum assembly of empirical knowledge about their biology and ecological features, the conditions for ecological understanding of the study system are limited. The “closed-reference” OTU approach was used in this study, not only to identify species in the target habitats, but also to exclude OTUs that were probably sedimented eDNA, originating in external habitats. By comparing species identifications from eDNA with species lists of morphologically identified species from the same samples, we could also assess some aspects of the detection efficiency of the two approaches.

Comparisons of morphology-identified taxa in each grab sample show relatively weak overlap among samples and that 40–70 (mean 48, std 10.3) % of the species recorded at one station were recorded uniquely from just one of the five grab samples. This clearly reflects that the animals are not evenly dispersed in the benthos. Such spatial distributions certainly have bearings on decisions about sampling regimes (see *e.g.*, Holte & Buhl-Mortensen, 2020). With eDNA one must additionally consider how biotechnological factors (Cowart et al., 2015) and informatic filtering procedures will affect diversity statistics (*e.g.*, Coissac, Riaz & Puillandre, 2012).

### Perspectives on OTU-filtering

From studies of microbiological communities based on 16S metabarcoding, it has become commonplace to group amplicons using a standard of minimum 97% similarity (Edgar, 2016, 2018; Holman et al., 2019; Singer et al., 2019; Porter & Hajibabaei, 2020) and to take OTUs as proxies of species. Similar ideas about group membership are also behind the concept of “bins” in Boldsystems (Ratnasingham & Hebert, 2013). By extension of this practice, fixed threshold values, often 97–98%, for similarity between OTU and labelled sequence in the reference database are used in species annotations of amplicons (*e.g.*, Alberdi et al., 2018; Descôteaux et al., 2021; Lacoursière-Roussel et al., 2018; Leray & Knowlton, 2016). However, many of the marine invertebrate species have proved to vary much more than 10% in the Folmer segment and a filtering pipeline that does not allow for intraspecific variability would potentially discard OTUs that are lacking exact sequence variants in the reference database. For example, a 98% cut-off would exclude about 35% of our annotated invertebrate OTUs, including the sequence reads of *Ciliatocardium ciliatum* (96.63% similar) and *Serripes groenlandicus* (92.19% similar), bivalve molluscs known to occur in Svalbard benthos. Thus, whilst metabarcoding is ideally aiming at species level resolution, it is often not possible in practice because matching annotated sequences are not found in reference databases (McGee, Robinson & Hajibabaei, 2019; Weigand et al., 2019). A stringent demand for exact sequence match would also amplify this problem of barcode deficiency, because species level annotations will require broad coverage of many, if not all, haplotypes that signify the species. Pragmatic considerations should allow for some flexibility in similarity tolerances while keeping in mind that public access reference libraries are developing products of scientific activities and not flawless fact files of identification engines.

While Boldigger search will report hits with similarity down to 50%, such low-end matches are usually uninformative even to the level of phylum. Rather than using only strict similarities for annotations, we initially applied a similarity threshold of 90% to identify candidate taxa. A relaxed similarity cut-off may increase the chance of detecting taxa that are not yet represented in reference libraries with exact sequence variants (Callahan, McMurdie & Holmes, 2017). For example, our amplicons showing 90% similarity to those reported as *Acharax* sp CST1 (Fukasawa et al., 2017) appear to be possible candidates of yet genetically uncharacterized *Acharax*, or at least a species of the family Solemyidae. This is particularly intriguing, because endemic *Acharax* associated with methane seeps off Svalbard (Hansen et al., 2019) have not yet been recorded from live specimens and accordingly not been DNA-sequenced. *Acharax* sp CST1 was found in the Pacific Chishima Trench (Fukasawa et al., 2017).

The LULU algorithm is designed to remove artefactual OTUs without discarding rare and real OTUs. It works by merging OTUs with sequence errors (daughter sequences) with more abundant ‘parent’ OTUs (Frøslev et al., 2017). We observed that this reduced the number of OTUs matching *T. longicaudata*, as intended. However, filtering with LULU also completely removed the few detections of *Scoletoma fragilis* and *Catablema vesicarium*, which also was an unintended effect. These results might indicate that the parameter settings of the LULU algorithm were not ideal. Brandt et al. (2021) found that for COI, more OTU clusters were retained using a minimum match of 90%, instead of 84% applied on our data. Thus, for future studies, researchers should ensure the parameter settings are appropriate for the genetic marker used.

Some automatic filtering strategies remove OTUs that are not consistently present in all sample replicates or retain only sequences that are present in at least *n* out of *m* PCR replicates (Alberdi et al., 2018). This is certainly not recommended if the aim of the research is to characterize communities that also include rare or low abundant organisms (Macher, Beermann & Leese, 2021). Similar exclusion of rare occurrence data may also come about if *relative abundance* cut-off values are employed in OTU filtering with the purpose to alleviate inflation of beta diversity estimates. These considerations are certainly important when eDNA analyses aim to target specific target organisms such as invasive or endangered species (Singer et al., 2019), and there is also a risk to exclude detections of functionally important community members (Leray & Knowlton, 2017).

### False positives

Metabarcode experiments have often focused on false positives generated from PCR errors and contaminations (Chambert, Miller & Nichols, 2015; Darling & Mahon, 2011; Darling et al., 2020; Ficetola et al., 2014, 2015; Ficetola, Taberlet & Coissac, 2016; Lahoz-Monfort, Guillera-Arroita & Tingley, 2016). We believe that we have addressed such problems in this work. False positives resulting from sample contamination may not necessarily be straightforward to pin down, even if they stand out as instances of taxa displaced from their natural habitat. We detected eDNA from photosynthesizing algae and from several terrestrial arthropods. There were also amplicons from fish, birds, and seals, signifying the presence of more or less peripheral non-resident organisms in the surrounding environments. Combined with qualified estimates of site specific eDNA degradation rates and oceanographic modelling, it could be possible to exploit such data to study transport and sedimentation of fine-particulate material (Collins et al., 2019). However, such exogenous eDNA appeals to some caution when interpreting biodiversity in benthic habitats. Because the benthos also is recipient of eDNA from the water column, it may be difficult to decide whether species detections represent genuine benthic occurrences or exogenous material from meroplanktonic life history stages (*e.g.*, Descôteaux et al., 2021). In these two Svalbard fjords, there are also possibilities of sediment disturbance and redistribution of eDNA from accidental events such as bottom trawling or glacier activity (Somerfield et al., 2006).

Taxonomic flaws in reference data (Radulovici et al., 2021) are also a potential source of false positives, particularly in understudied marine organisms. Too relaxed similarity thresholds for taxonomic annotation may certainly contribute to false positives and decisions must somehow be balanced against the level of required taxonomic resolution. High stringency is certainly required when particular species detections form the fundament for management decisions.

DNA-studies have uncovered unexpectedly high genetic diversity within nominal species that have traditionally been recognized from morphological characteristics. Genetic differences or non-monophyly are challenging traditional concepts of species. Many marine invertebrate groups are therefore presently in a state of taxonomic flux, as new species discoveries are awaiting formal description and others are awaiting revision. The international DNA-barcode campaign and Boldsystems.org have also exposed frequent discordance amongst identifiers in their taxonomic interpretation of nominal species. This is well demonstrated by many cases of Bins in BOLD, having similar sequences but two or more species names. Such conflicts were also disclosed by our Blastn search, when BOLD and GenBank returned different species identifications of the same sequence (Table 1). Because some of our identifications prompted special attention, we refer to Supplementary Material (Text S1) for taxonomic comments.

### False negatives

In general, appraisals of biodiversity should take into consideration that the target units may remain undetected, despite being present in the study area (Ficetola et al., 2015; Roussel et al., 2015; Beng & Corlett, 2020; Tzafesta et al., 2021). While this understanding was certainly developed before eDNA surveys, biodiversity assessment based on eDNA has added some extra dimensions to the types of potentially erroneous observations. One set of issues is associated with the “ecology” of extra-organismal DNA (Barnes & Turner, 2016; Collins et al., 2018; Harrison, Sunday & Rogers, 2019; Lacoursière-Roussel & Deiner, 2019; Holman, Chng & Rius, 2022), how its origin, transport, biochemistry, and degradation is affected by environmental factors. Another set of problems is rooted in taxonomy, our yet limited understanding of the units of biodiversity in marine environments, incomplete DNA-barcode archives, and different methodological practices among research groups.

We noticed that several of the species that were identified with traditional sample sorting and identification were not detected in our DNA-material (Figs. 7 and 8). For polychaetes the proportion of DNA-discovered species per station was 17–47% (mean 20.6%) of the morphology identified species. These are relatively high numbers, as Aylagas et al. (2016) detected only about 3% of the morphologically identified species in their sediment eDNA samples, and Staehr et al. (2022) only 13% of diver-detected macroalgae. Our data also indicated that about 13% morphology identified taxa were also identified with DNA. A recent meta-analysis of eDNA metabarcoding *vs* traditional methods Keck et al. (2022) also found pronounced differences in taxa composition between the two approaches, particularly with respect to invertebrates, plankton, and microphytobenthos. In our comparisons, we ascribe a small proportion of the divergent taxonomic composition of morphology *vs* DNA-identified material (Fig. 7) to different taxonomic resolutions in the two sets of data. This is because morphologically identified higher level taxa and species level identifications may have to be counted as different units. Such methodical bias should logically favour higher diversities in DNA based taxa counts that are based on species. When traditional identifications recorded more taxa than metabarcoding, larger sample volumes (wider microhabitats) and sorting efforts probably contributed to our higher diversity estimates. We sampled totally only about 2% of the grab surface volume that was examined by manual sorting.

Casey et al. (2021) have shown how the choice of genetic barcode marker for metabarcoding can produce a skewed picture of metazoan diversity. A particularly striking example of this was our detection of only two species of nematodes in Kongsfjord, whereas van den Heuvel-Greve et al. (2021) detected 33 species using 18S primers, but only one with CO1. Even 33 may seem like a small number, considering the proposition that Kongsfjord has more than hundred species of nematodes as identified by morphology (Somerfield et al., 2006).

If the similarity threshold for acceptance is high and a particular haplotype is not present in the reference database, a species may be overlooked despite it being represented with other haplotypes in the database. We tried to address this potential problem by accepting a relatively tolerant (90%) similarity threshold. However, many of the relevant target sequences were produced from regional collections, suggesting reasonably good representation of undetected polychaetes in the database. We also observed that most of the species that were not detected with DNA were indeed represented in the search database.

### Diversity and ecological observations

Our estimates of Chao1 from eDNA had standard errors (Fig. 6) from 0 to maximum 13.64, in the latter case with exceptional estimates of 15 infauna species more than observed in one of the replicates from station R1881 (Fig. 6). However, especially low richness counts usually had the same estimates with Chao1. Shannon¨s index, like Chao1 incorporates abundances, which in this context means numbers of sequence reads. We did not observe any relationship between species abundance/weight and sequence reads (not shown, however see Figs. 8 and S5). Whether such correlative relations can be established in invertebrate community studies is an open question, and currently a bottleneck in attempts to tune metabarcoding to traditional quantitative macroinvertebrate studies for monitoring (Deiner et al., 2017; van der Loos & Nijland, 2021). Against a backdrop of uncertainties associated with DNA shedding, transport and preservation, PCR bias and other technical and statistical challenges, some caution is advised when levelling sequence reads with abundance of individuals. Shannon’s index showed several examples of values near zero (Fig. 6) representing 1–3 species in one sample and similar to the dispersed pattern seen in morphology identified species (Fig. 5). Except the polychaetes, our sampling gave a picture of a relatively moderate species diversity of benthic invertebrates, and it is somewhat surprising that so few species of molluscs, crustaceans, and echinoderms were detected. Mean species richness between the fjords was relatively similar. Underlying differences in species composition was revealed by the PCoA, which expressed a significant separation of the two fjord systems based on presence-absence from both data sets. The correspondence among data points from the two sets was reasonably good on the first axis, as also indicated by the ordination centroids. The centroids for the second axes and the clusters of taxa from station R1929 (Fig. 9) placed at opposite ends of the second axis, prompted the idea that one of the taxa-ordinations could be rotated 180 degrees on the TOC-vector for a better visual fit between the two ordinations. Both fits showed the percentage of sand in the sediments as the most influential on PC-axis 2. Apart from geography and bottom depth, the highest scores on the first axis produced from the DNA-data were total organic matter and organic carbon. The morphology- based data had TOC as the highest scores (Fig. 9, Table S7). Apart from the geographical separation of the fjords, Kongsfjord sites were also deeper than those from Rijpfjord. This was reflected in the ordination and in the fit of the environment vectors (Fig. 9, Table S7). The differences between these fjord systems may also have to be considered in the perspective of anthropogenic influence. MAREANO video inspections show that Kongsfjord has sometimes dense visible marks of trawling activities, whereas Rijpfjord seems undisturbed by trawls (interactive map at https://tinyurl.com/4f4c5mwd). One noticeable biological observation is the relative abundance of sponges in Rijpfjord, where the diversity contrast between the southern sites and the northern one (Figs. 1 and 6) was also exposed by the PERMANOVA analysis. Sponges were particularly abundant at the northern site (R1929), which is a more open locality than the inner fjord sites, and closer to the ice edge. The site has slightly more organic carbon than the southern sites, but still less than most of the Kongsfjord sites. The contrast to station R1887, where polychaetes dominated the read abundance, was particularly apparent (Fig. 4).

We notice that, despite the relatively weak taxon overlap between the DNA-based and the morphology-based identifications, there is considerable congruence in the structural patterns displayed by principal coordinate ordination of each data set.

## Conclusions and Recommendations

When metabarcoding marine benthic samples, we found that only about 50% of the raw OTUs returned 50% or better similarity hits from search in the BOLD database, indicating missing reference sequences in the CO1 database. Because many invertebrates exhibit intraspecific differences of more than 3%, accepting only exact sequence variants (Callahan, McMurdie & Holmes, 2017) or those with more than 97% similarity reduce taxa detection rates. We set a lower limit of 90% similarity between subject and hit sequence to detect candidate taxa that may not yet have exact sequence variants in the reference library. The Polychaeta dominated the amplicon abundance with about 75% of the sequence reads, followed by Nematoda. We observed that only 17% to 47% of the polychaete species identified with morphology were also detected with DNA. We found that 90–100% (mean 96.7%) of these species identified from morphology are represented with CO1 barcodes in Boldsystems, so a majority of missing species detections were not due to database gaps.

We also observed several examples of primer/multi-template mismatches that could potentially contribute amplification bias or false negatives. However, our summed replicate samples for metabarcoding are covering only about 2% of the sample space examined by visual sorting and identification. Because macro-invertebrates often have scattered or clumped distributions in the sediments, many species may remain undetected with metabarcoding, depending on the extent of the sampling regime. Increasing sample size or replicates may increase species detection rates but will also increase processing efforts and expenses.

Metabarcoding detected a considerable proportion of taxa that were not recorded from visual sorting, many of which are meiofauna, small epibenthos or extracellular DNA from pelagic species. The ability to detect and identify meio- and microfauna broadens the repertoire of routine benthic monitoring (Lanzén et al., 2021), and censusing of species diversity. But sedimented, exogenous DNA may contribute to PCR-bias and to unknown effects in taxon-free assessments of biotic diversity. Unless the influx of exogenous matter is the focal point of the study, amplicons from exogenous organisms should be removed for *in situ* habitat analyses. If OTUs are identified, removal of some taxa may sometimes be trivial tasks. However, as is the case with bentho-pelagic taxa, this may sometimes be difficult. Accordingly, when metabarcoding detects more species than a traditional approach it will be interesting to know the proportion that represents non-residential organisms.

Despite some of the shortages identified in this study, metabarcoding will complement traditional analyses of benthic biodiversity. However, this work and several other analyses (Cahill et al., 2018; Pawlowski et al., 2021; Keck et al., 2022) indicate that assessments of invertebrate species diversity based on metabarcoding may considerably differ from those obtained from visual identifications. For practical applications in bioassessment and ecosystem management, it is important to know the stronger and weaker sides of both methodologies.

We noticed several examples of taxonomic discordance in sequence records and published literature. Such discoveries will trigger revisionary taxonomic work, help to standardize identifications, and improve our knowledge of biodiversity in comparisons across regions and research communities. Because DNA barcoding and metabarcoding combine species inventory with publicly available information systems, these methodologies are also invaluable tools to discover, report, analyse, and globally harmonize the organism taxonomies that underpin ecology and biodiversity science.

The number of sampling replicates is probably more important than sample volumes and must be scaled to the purpose of the project. Denser sampling should increase the detection probability of rare or widely dispersed species. An increased number of sample replicates, with addition of more markers than CO1, may increase detection efficiency, but ultimately lead to loss of the financial and time effective advantages metabarcoding is supposed to have over traditional methods. If the purpose of the census is to monitor rare/invasive/threatened taxa, they are better targeted with special discriminating primers.

A better understanding of quantitative relationships (if any) between sequence reads, numbers of individuals, biomass and other population key parameters must be developed to either replace or integrate metabarcoding with traditional monitoring programs. Although identification from barcodes has opened for quick “snapshots” of species richness, abundance-based bioindicators, biomass, and production estimates of central taxa groups in the ecosystem are also important for management.

Specifically for monitoring Arctic marine systems that are currently influenced by climate change and increased human impacts, monitoring needs time series that are based on standardized methodologies (*e.g.*, CAFF, 2011). Given the diverse set of biotechnological factors that may currently influence the outcomes of a study of marine communities based on metabarcoding (*e.g.*, van der Loos & Nijland, 2021), it seems wise to explore the *pros* and *cons* of new methodologies while also maintaining the best of the arts of morphology-methods.

## Supplemental Information

10.7717/peerj.14321/supp-1 Supplemental Information 1 Identifications with >90% similarities to objects in Boldsystems.org from surface (0–0.5 cm) and infauna (0–5 cm) grab samples with taxonomic comments.

10.7717/peerj.14321/supp-2 Supplemental Information 2 Invertebrate taxa identified with >90% similarity hits from the top layers (0–0.5 cm).

10.7717/peerj.14321/supp-3 Supplemental Information 3 Invertebrate taxa identified with >90% similarity hits from 0–5 cm sediment depth.

10.7717/peerj.14321/supp-4 Supplemental Information 4 Raw data from metabarcoding sediment samples from 0–0.5 cm depth.

10.7717/peerj.14321/supp-5 Supplemental Information 5 Raw data from metabarcoding sediment samples from 0–5 cm depth.

10.7717/peerj.14321/supp-6 Supplemental Information 6 Overview of the findings in the negative controls.

10.7717/peerj.14321/supp-7 Supplemental Information 7 Scores of environmental variables to axes PC1 and PC2 of PCo analysis of taxa occurrence data from eDNA and morphologically identified taxa. Also see Fig. 9.

10.7717/peerj.14321/supp-8 Supplemental Information 8 Results from the PERMANOVA tests based Hellinger transformed Bray-Curtis dissimilarity matrix.

10.7717/peerj.14321/supp-9 Supplemental Information 9 Approaches to analyses of Svalbard benthos: Traditional sorting and identification *vs* metabarcoding based on prior barcoding of fauna to reference database.

10.7717/peerj.14321/supp-10 Supplemental Information 10 Taxonomic composition of pooled >90% similarity reads identified with Boldigger from 0–0.5 cm sediment surface samples from Kongsfjord and Rijpfjord, Svalbard.

10.7717/peerj.14321/supp-11 Supplemental Information 11 Taxonomic composition of pooled >90% similarity reads identified with Boldigger from 0–5 cm sediment infauna samples (0–5 cm) from Kongsfjord and Rijpfjord, Svalbard.

10.7717/peerj.14321/supp-12 Supplemental Information 12 Distribution of numbers of 5′ primer mismatches in sequences from detected and undetected species of Polychaeta.

10.7717/peerj.14321/supp-13 Supplemental Information 13 Numbers of individuals of each visually identified polychaete species vs numbers of 5’-primer mismatches in CO1 sequences of those species.

10.7717/peerj.14321/supp-14 Supplemental Information 14 Taxonomic remarks.

## Additional Information and Declarations

### Field Study Permissions

The following information was supplied relating to field study approvals (*i.e.*, approving body and any reference numbers): Field collections were approved by the Govenor of Svalbard.

### Data Availability

The following information was supplied regarding data availability: The raw data from metabarcoding and the MOTUs from the OBITools pipeline with the corresponding sequences are available in the Supplemental Files.
