# Supplementary material for: Benthic invertebrates in Svalbard fjords—when metabarcoding does not outperform traditional biodiversity assessment
Source: PeerJ. 2022 Nov 17;10:e14321. doi: 10.7717/peerj.14321 (PMC9676020; doi:10.7717/peerj.14321)
Supplement: Supplemental Information 2 — Invertebrate taxa identified with >90% similarity hits from the top layers (0–0.5 cm). [file peerj-10-14321-s002.docx]

**SupTab 2 invertebrate taxa identified with >90 % similarity hits from the top layers (0-0.5 cm)**

| **Phylum** | **Class** | **Order** | **Family** | **Genus** | **Species** |
| --- | --- | --- | --- | --- | --- |
| Annelida | Polychaeta | Capitellida | Arenicolidae | *Arenicola* | *Arenicola marina* |
| Annelida | Polychaeta | Eunicida | Lumbrineridae | *Lumbrineris* | *Lumbrineris mixochaeta* |
| Annelida | Polychaeta | NI | Capitellidae | *Heteromastus* | *Heteromastus filiformis* |
| Annelida | Polychaeta | NI | Maldanidae | *Maldane* | *Maldane sp.* |
| Annelida | Polychaeta | NI | Maldanidae | *Microclymene* | *Microclymene sp.* |
| Annelida | Polychaeta | NI | Maldanidae | *Praxillella* | *Praxillella sp.* |
| Annelida | Polychaeta | Phyllodocida | Hesionidae | *Gyptis* | *Gyptis golikovi* |
| Annelida | Polychaeta | Phyllodocida | Nephtyidae | *Aglaophamus* | *Aglaophamus malmgreni* |
| Annelida | Polychaeta | Phyllodocida | Pholoidae | *Pholoe* | *Pholoe assimilis* |
| Annelida | Polychaeta | incertae sedis | Cossuridae | *Cossura* | *Cossura pygodactylata* |
| Annelida | Polychaeta | incertae sedis | Orbiniidae | *Leitoscoloplos* | *Leitoscoloplos mammosus* |
| Annelida | Polychaeta | incertae sedis | Orbiniidae | *Leitoscoloplos* | *Leitoscoloplos pugettensis* |
| Annelida | Polychaeta | Sabellida | Oweniidae | *Galathowenia* | *Galathowenia oculata* |
| Annelida | Polychaeta | Spionida | Spionidae | *Laonice* | *Laonice cirrata* |
| Annelida | Polychaeta | Spionida | Spionidae | NI | NI |
| Annelida | Polychaeta | Spionida | Spionidae | *Prionospio* | *Prionospio cirrifera* |
| Annelida | Polychaeta | Spionida | Spionidae | *Spio* | *Spio filicornis* |
| Annelida | Polychaeta | Terebellida | Cirratulidae | *Chaetozone* | *Chaetozone setosa* |
| Annelida | Polychaeta | Terebellida | Cirratulidae | *Chaetozone* | *Chaetozone sp.* |
| Annelida | Polychaeta | Terebellida | Terebellidae | *Polycirrus* | *Polycirrus arcticus* |
| Annelida | Polychaeta | Terebellida | Trichobranchidae | *Terebellides* | *Terebellides* |
| Arthropoda | Copepoda | Calanoida | Acartiidae | *Acartia* | *Acartia longiremis* |
| Arthropoda | Copepoda | Calanoida | Calanidae | *Calanus* | *Calanus glacialis* |
| Arthropoda | Copepoda | Calanoida | Clausocalanidae | *Microcalanus* | *Microcalanus pusillus* |
| Arthropoda | Copepoda | Harpacticoida | Ectinosomatidae | *Bradya* | *Bradya typica* |
| Arthropoda | Malacostraca | Amphipoda | NI | NI | NI |
| Arthropoda | Thecostraca | Sessilia | Balanidae | *Balanus* | *Balanus balanus* |
| Bryozoa | Gymnolaemata | Cheilostomatida | Candidae | *Tricellaria* | *Tricellaria ternate* |
| Chordata | Ascidiacea | Phlebobranchia | Ascidiidae | *Ascidia* | *Ascidia callosa* |
| Chordata | Ascidiacea | Stolidobranchia | Pyuridae | *Halocynthia* | *Halocynthia pyriformis* |
| Cnidaria | Hydrozoa | Anthoathecata | Boreohydridae | *Plotocnide* | *Plotocnide borealis* |
| Cnidaria | Hydrozoa | Anthoathecata | Pandeidae | *Catablema* | *Catablema vesicarium* |
| Cnidaria | Hydrozoa | Anthoathecata | Pandeidae | *Halitholus* | *Halitholus cirratus* |
| Cnidaria | Hydrozoa | Leptothecata | Lafoeidae | *Lafoea* | *Lafoea dumosa* |
| Cnidaria | Scyphozoa | Semaeostomeae | Cyaneidae | *Cyanea* | *Cyanea sp.* |
| Cnidaria | Scyphozoa | Semaeostomeae | Ulmaridae | *Aurelia* | *Aurelia aurita* |
| Cnidaria | Staurozoa | Stauromedusae | Lucernariidae | *Lucernaria* | *Lucernaria bathyphila* |
| Echinodermata | Asteroidea | Paxillosida | Ctenodiscidae | *Ctenodiscus* | *Ctenodiscus crispatus* |
| Echinodermata | Echinoidea | Camarodonta | Strongylocentrotidae | *Strongylocentrotus* | *Strongylocentrotus droebachiensis* |
| Echinodermata | Ophiuroidea | Amphilepidida | Ophiactidae | *Ophiopholis* | *Ophiopholis aculeata* |
| Echinodermata | Ophiuroidea | Ophiurida | Ophiuridae | *Ophiura* | *Ophiura albida* |
| Mollusca | Bivalvia | Adapedonta | Hiatellidae | *Hiatella* | *Hiatella sp.* |
| Mollusca | Bivalvia | Nuculanida | Yoldiidae | *Yoldiella* | *Yoldiella frigida* |
| Mollusca | Bivalvia | Solemyida | Solemyidae | *Acharax* | *Acharax sp.* |
| Nematoda | Chromadorea | Monhysterida | Linhomoeidae | *Terschellingia* | *Terschellingia longicaudata* |
| Nematoda | Chromadorea | Monhysterida | Sphaerolaimidae | *Parasphaerolaimus* | *Parasphaerolaimus paradoxus* |
| Nemertea | Hoplonemertea | Monostilifera | NI | NI | Monostilifera sp. |
| Nemertea | Palaeonemertea | NI | Cephalothricidae | *Cephalothrix* | *Cephalothrix iwatai* |
| Porifera | NI | NI | NI | NI | NI |
| Priapulida | Priapulimorpha | Priapulimorphida | Priapulidae | *Priapulus* | *Priapulus caudatus* |
