# Supplementary material for: Benthic invertebrates in Svalbard fjords—when metabarcoding does not outperform traditional biodiversity assessment
Source: PeerJ. 2022 Nov 17;10:e14321. doi: 10.7717/peerj.14321 (PMC9676020; doi:10.7717/peerj.14321)
Supplement: Supplemental Information 3 — Invertebrate taxa identified with >90% similarity hits from 0–5 cm sediment depth. [file peerj-10-14321-s003.docx]

**SupTab 3 invertebrate taxa identified with >90 % similarity hits from 0-5 cm sediment depth**

| **Phylum** | **Class** | **Order** | **Family** | **Genus** | **Species** |
| --- | --- | --- | --- | --- | --- |
| Annelida | Polychaeta | Eunicida | Lumbrineridae | *Abyssoninoe* | *Abyssoninoe sp.* |
| Annelida | Polychaeta | Eunicida | Lumbrineridae | *Lumbrineris* | *Lumbrineris mixochaeta* |
| Annelida | Polychaeta | Eunicida | Lumbrineridae | *Scoletoma* | *Scoletoma fragilis* |
| Annelida | Polychaeta | NI | Capitellidae | *Heteromastus* | *Heteromastus filiformis* |
| Annelida | Polychaeta | NI | Maldanidae | *Eupraxillella* | *Eupraxillella sp.* |
| Annelida | Polychaeta | NI | Maldanidae | *Lumbriclymene* | *Lumbriclymene sp.* |
| Annelida | Polychaeta | NI | Maldanidae | *Maldane* | *Maldane sarsi* |
| Annelida | Polychaeta | NI | Maldanidae | *Maldane* | *Maldane sp.* |
| Annelida | Polychaeta | NI | Maldanidae | *Microclymene* | *Microclymene sp.* |
| Annelida | Polychaeta | NI | Maldanidae | *Nicomache* | *Nicomache lumbricalis* |
| Annelida | Polychaeta | NI | Maldanidae | *Nicomache* | *Nicomache minor* |
| Annelida | Polychaeta | Phyllodocida | Hesionidae | *Gyptis* | *Gyptis golikovi* |
| Annelida | Polychaeta | Phyllodocida | Pholoidae | *Pholoe* | *Pholoe assimilis* |
| Annelida | Polychaeta | Phyllodocida | Phyllodocidae | *Eteone* | *Eteone* |
| Annelida | Polychaeta | Phyllodocida | Phyllodocidae | *Eteone* | *Eteone cf. flava* |
| Annelida | Polychaeta | Incertae sedis | Cossuridae | *Cossura* | *Cossura longocirrata* |
| Annelida | Polychaeta | Incertae sedis | Cossuridae | *Cossura* | *Cossura pygodactylata* |
| Annelida | Polychaeta | Incertae sedis | Orbiniidae | *Leitoscoloplos* | *Leitoscoloplos pugettensis* |
| Annelida | Polychaeta | Incertae sedis | Orbiniidae | *Scoloplos* | *Scoloplos sp.* |
| Annelida | Polychaeta | Incertae sedis | Paraonidae | *Aricidea* | *Aricidea quadrilobata* |
| Annelida | Polychaeta | Sabellida | Owenidae | *Galathowenia* | *Galathowenia oculata* |
| Annelida | Polychaeta | Sabellida | Sabellidae | NI | NI |
| Annelida | Polychaeta | Spionida | Spionidae | *Laonice* | *Laonice cirrata* |
| Annelida | Polychaeta | Spionida | Spionidae | NI | NI |
| Annelida | Polychaeta | Spionida | Spionidae | *Prionospio* | *Prionospio cirrifera* |
| Annelida | Polychaeta | Terebellida | Cirratulidae | *Aphelochaeta* | *Aphelochaeta sp.* |
| Annelida | Polychaeta | Terebellida | Cirratulidae | *Chaetozone* | *Chaetozone* |
| Annelida | Polychaeta | Terebellida | Cirratulidae | *Chaetozone* | *Chaetozone setosa* |
| Annelida | Polychaeta | Terebellida | Cirratulidae | *Chaetozone* | *Chaetozone sp.* |
| Annelida | Polychaeta | Terebellida | Cirratulidae | *Dodecaceria* | *Dodecaceria concharum* |
| Annelida | Polychaeta | Terebellida | Terebellidae | *Laphania* | *Laphania boecki* |
| Annelida | Polychaeta | Terebellida | Terebellidae | *Polycirrus* | *Polycirrus arcticus* |
| Arthropoda | Copepoda | Calanoida | Acartiidae | *Acartia* | *Acartia* |
| Arthropoda | Copepoda | Calanoida | Calanidae | *Calanus* | *Calanus sp.* |
| Arthropoda | Copepoda | Calanoida | Clausocalanidae | *Microcalanus* | *Microcalanus pusillus* |
| Arthropoda | Copepoda | Calanoida | Clausocalanidae | *Pseudocalanus* | *Pseudocalanus acuspes* |
| Arthropoda | Copepoda | Calanoida | Clausocalanidae | *Pseudocalanus* | *Pseudocalanus sp.* |
| Arthropoda | Malacostraca | Amphipoda | Pleustidae | *Pleusymtes* | *Pleusymtes glaber* |
| Arthropoda | Thecostraca | Sessilia | Balanidae | *Balanus* | *Balanus balanus* |
| Bryozoa | Gymnolaemata | Cheilostomatida | Candidae | *Scrupocellaria* | *Scrupocellaria sp.* |
| Bryozoa | Gymnolaemata | NI | NI | NI | NI |
| Chordata | Ascidiacea | Phlebobranchia | Ascidiidae | *Ascidia* | *Ascidia callosa* |
| Chordata | Ascidiacea | Stolidobranchia | Pyuridae | *Boltenia* | *Boltenia echinata* |
| Chordata | Ascidiacea | Stolidobranchia | Pyuridae | *Halocynthia* | *Halocynthia pyriformis* |
| Cnidaria | Anthozoa | Actiniaria | Halcampidae | *Halcampa* | *Halcampa sp.* |
| Cnidaria | Hydrozoa | Anthoathecata | Boreohydridae | *Plotocnide* | *Plotocnide borealis* |
| Cnidaria | Hydrozoa | Anthoathecata | Corynidae | *Sarsia* | *Sarsia princeps* |
| Cnidaria | Hydrozoa | Leptothecata | Lafoeidae | *Lafoea* | *Lafoea dumosa* |
| Cnidaria | Hydrozoa | Leptothecata | Sertulariidae | *Symplectoscyphus* | *Symplectoscyphus tricuspidatus* |
| Cnidaria | Scyphozoa | Semaeostomeae | Cyaneidae | *Cyanea* | *Cyanea sp.* |
| Cnidaria | Staurozoa | Stauromedusae | Lucernariidae | *Lucernaria* | *Lucernaria bathyphila* |
| Echinodermata | Asteroidea | Paxillosida | Ctenodiscidae | *Ctenodiscus* | *Ctenodiscus crispatus* |
| Echinodermata | Echinoidea | Camarodonta | Strongylocentrotidae | *Strongylocentrotus* | *Strongylocentrotus droebachiensis* |
| Echinodermata | Echinoidea | Camarodonta | Strongylocentrotidae | *Strongylocentrotus* | *Strongylocentrotus pallidus* |
| Echinodermata | Holothuroidea | Molpadida | Molpadiidae | *Molpadia* | *Molpadia borealis* |
| Echinodermata | Ophiuroidea | Ophiacanthida | Ophiacanthidae | *Ophiacantha* | *Ophiacantha bidentata* |
| Kinorhyncha | Incertae sedis | Cyclorhagida | Echinoderidae | *Echinoderes* | *Echinoderes svetlanae* |
| Mollusca | Bivalvia | Cardiida | Cardiidae | *Ciliatocardium* | *Ciliatocardium ciliatum* |
| Mollusca | Bivalvia | Cardiida | Cardiidae | *Serripes* | *Serripes groenlandicus* |
| Mollusca | Bivalvia | Lucinida | Thyasiridae | *Adontorhina* | *Adontorhina* |
| Mollusca | Bivalvia | Nuculanida | Yoldiidae | *Yoldiella* | *Yoldiella cf. frigida* |
| Mollusca | Bivalvia | Nuculanida | Yoldiidae | *Yoldiella* | *Yoldiella frigida* |
| Mollusca | Bivalvia | Solemyida | Solemyidae | *Acharax* | *Acharax sp.* |
| Mollusca | Solenogastres | NI | Simrothiellidae | *Plawenia* | *Plawenia* |
| Nematoda | Chromadorea | Monhysterida | Linhomoeidae | *Terschellingia* | *Terschellingia longicaudata* |
| Nematoda | Chromadorea | Monhysterida | Sphaerolaimidae | *Parasphaerolaimus* | *Parasphaerolaimus paradoxus* |
| Nemertea | Hoplonemertea | Monostilifera | NI | NI | Monostilifera sp. |
| Nemertea | Palaeonemertea | NI | Cephalothricidae | *Cephalothrix* | *Cephalothrix iwatai* |
| Platyhelminthes | Turbellaria | Acoela | Haploposthiidae | *Haploposthia* | *Haploposthia rubra* |
| Porifera | Demospongiae | Poecilosclerida | Acarnidae | *Iophon* | *Iophon sp.* |
| Porifera | Demospongiae | Suberitida | Suberitidae | *Plicatellopsis* | *Plicatellopsis bowerbanki* |
| Porifera | NI | NI | NI | NI | NI |
| Sipuncula | Sipunculidea | Golfingiida | Golfingiidae | *Golfingia* | *Golfingia margaritacea* |
