# Supplementary material for: Benthic invertebrates in Svalbard fjords—when metabarcoding does not outperform traditional biodiversity assessment
Source: PeerJ. 2022 Nov 17;10:e14321. doi: 10.7717/peerj.14321 (PMC9676020; doi:10.7717/peerj.14321)
Supplement: Supplemental Information 9 — Approaches to analyses of Svalbard benthos: Traditional sorting and identification vs metabarcoding based on prior barcoding of fauna to reference database. [file peerj-10-14321-s009.pdf]

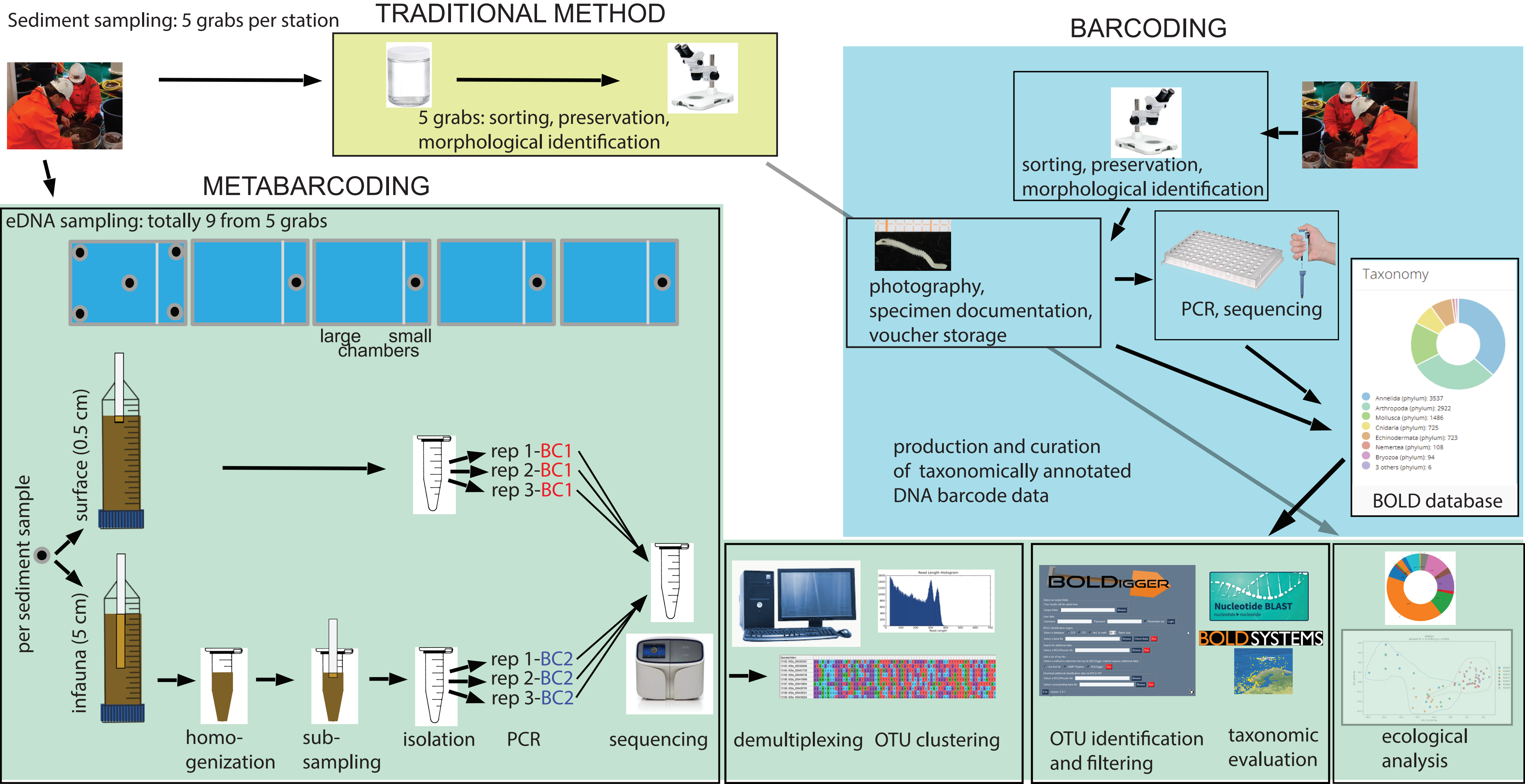
Sediment sampling: 5 grabs per station
TRADITIONAL METHOD
5 grabs: sorting, preservation, morphological identification
BARCODING
sorting, preservation, morphological identification
photography, specimen documentation, voucher storage
PCR, sequencing
Taxonomy
Annelida (phylum): 3537
Arthropoda (phylum): 2922
Mollusca (phylum): 1486
Cnidaria (phylum): 725
Echinodermata (phylum): 723
Nemertea (phylum): 108
Bryozoa (phylum): 94
3 others (phylum): 6
BOLD database
production and curation of taxonomically annotated DNA barcode data
METABARCODING
eDNA sampling: totally 9 from 5 grabs
large small chambers
per sediment sample
surface (0.5 cm)
infauna (5 cm)
rep 1-BC1
rep 2-BC1
rep 3-BC1
rep 1-BC2
rep 2-BC2
rep 3-BC2
homo-genization
sub-sampling
isolation
PCR
sequencing
demultiplexing
OTU clustering
BOLDigger
Nucleotide BLAST
BOLDSYSTEMS
OTU identification and filtering
taxonomic evaluation
ecological analysis
