# Supplementary figures and images for: Benthic invertebrates in Svalbard fjords—when metabarcoding does not outperform traditional biodiversity assessment
Source: PeerJ. 2022 Nov 17;10:e14321. doi: 10.7717/peerj.14321 (PMC9676020; doi:10.7717/peerj.14321)

779427

% occupancy: 35 5378411191811

## surface samples

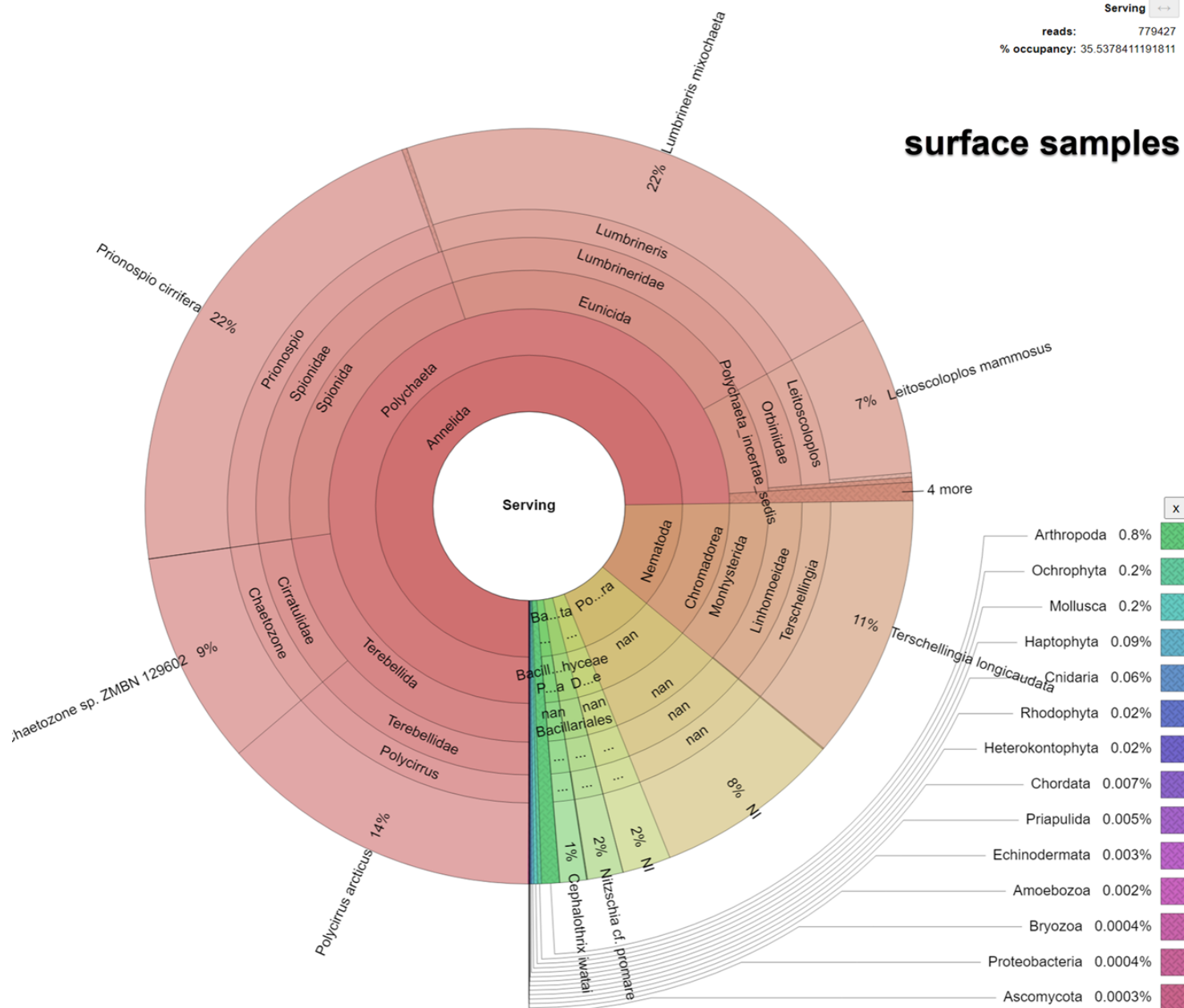

Supplement: Supplemental Information 10 — Taxonomic composition of pooled >90% similarity reads identified with Boldigger from 0–0.5 cm sediment surface samples from Kongsfjord and Rijpfjord, Svalbard. [file peerj-10-14321-s010.pdf]

# infauna

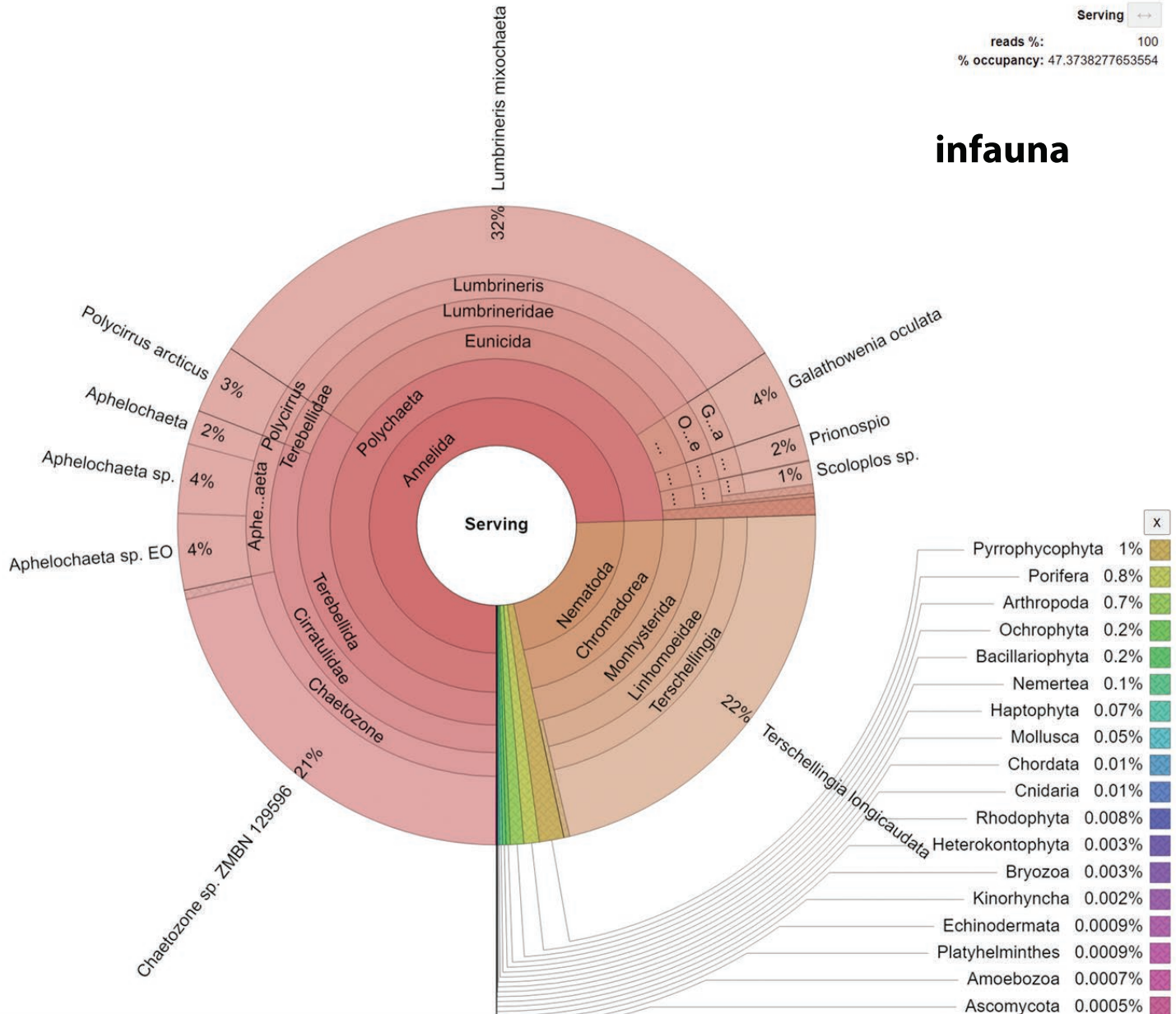

Supplement: Supplemental Information 11 — Taxonomic composition of pooled >90% similarity reads identified with Boldigger from 0–5 cm sediment infauna samples (0–5 cm) from Kongsfjord and Rijpfjord, Svalbard. [file peerj-10-14321-s011.pdf]

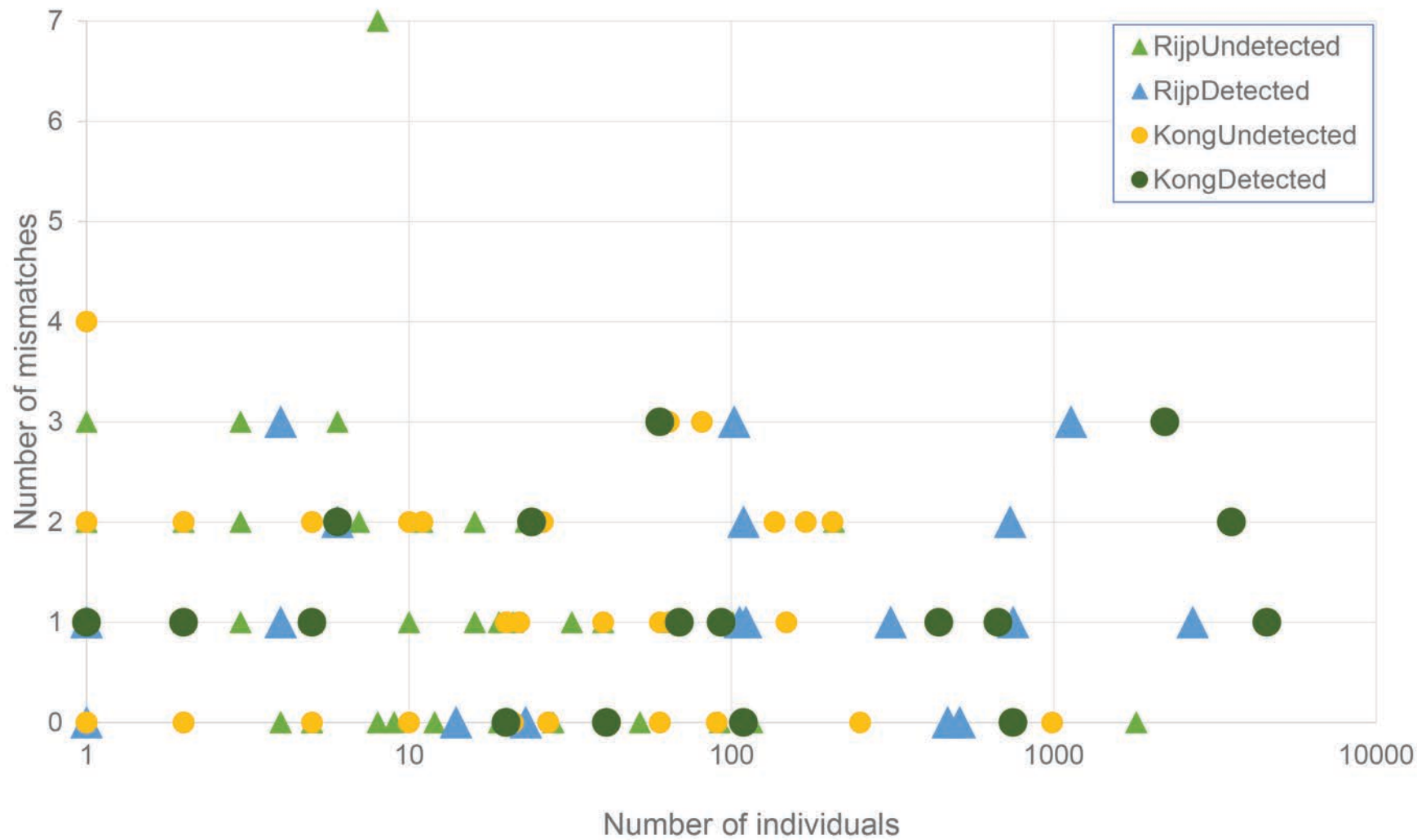

Supplement: Supplemental Information 13 — Numbers of individuals of each visually identified polychaete species vs numbers of 5’-primer mismatches in CO1 sequences of those species. [file peerj-10-14321-s013.pdf]
