# Supplementary material for: Benthic invertebrates in Svalbard fjords—when metabarcoding does not outperform traditional biodiversity assessment
Source: PeerJ. 2022 Nov 17;10:e14321. doi: 10.7717/peerj.14321 (PMC9676020; doi:10.7717/peerj.14321)
Supplement: Supplemental Information 12 — Distribution of numbers of 5′ primer mismatches in sequences from detected and undetected species of Polychaeta. [file peerj-10-14321-s012.pdf]

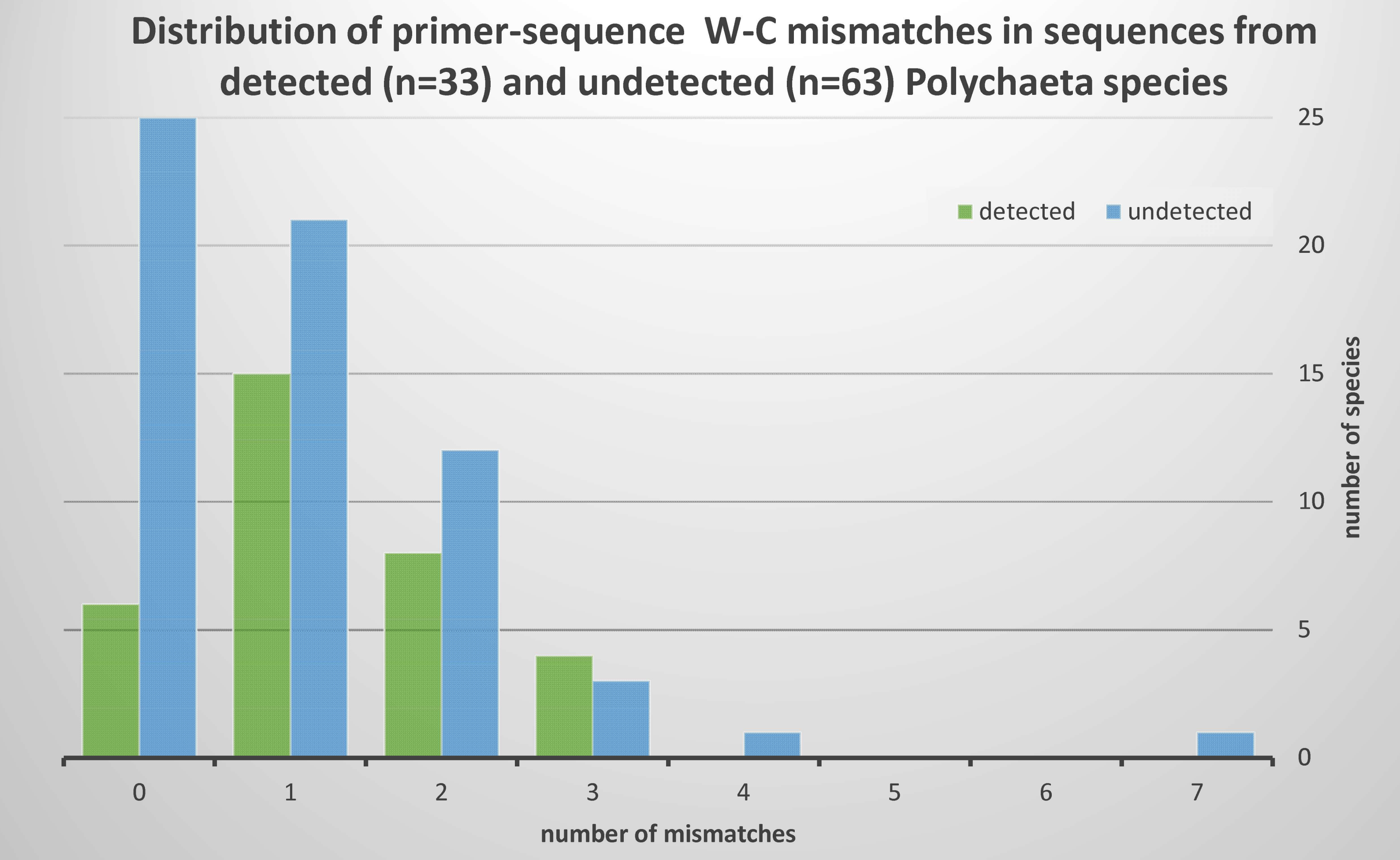

Distribution of primer-sequence W-C mismatches in sequences from detected (n=33) and undetected (n=63) Polychaeta species
detected
undetected
25
20
15
10
5
0
number of species
0
1
2
3
4
5
6
7
number of mismatches
