# Supplementary material for: Benthic invertebrates in Svalbard fjords—when metabarcoding does not outperform traditional biodiversity assessment
Source: PeerJ. 2022 Nov 17;10:e14321. doi: 10.7717/peerj.14321 (PMC9676020; doi:10.7717/peerj.14321)
Supplement: Supplemental Information 14 — Taxonomic remarks. [file peerj-10-14321-s014.docx]

**Supplement text S1**

Endre Willassen^1^, Jon-Ivar Westgaard^2^, Jon Anders Kongsrud^1^, Tanja Hanebrekke^2^, Pål Buhl-Mortensen^3^, Børge Holte^4^

Benthic invertebrates in Svalbard fjords. - When metabarcoding does not outperform traditional biodiversity assessment

**Taxonomic remarks**

The polychaete *Chaetozone setosa*, which has been regarded as a bioindicator of “slight to pronounced unbalanced situations” (Borja et al., 2000; Teixeira et al., 2016) in ecological assessment schemes, is a telling example of taxa that have been revised following DNA studies. Grosse et al. (2020, 2021) found that *Chaetozone setosa* is a complex of cryptic species and have recently provided some of those that are still marked as *C. setosa* in BOLD with new names. Sequences in BOLD:ACZ4936 should now refer to *Chaetozone* *pseudosetosa* and BOLD:ACV1642 to *Chaetozone* *monteverdii*. We detected what is believed to be the genuine *C. setosa* (Grosse et al., 2021), and which was originally described from Svalbard and corresponds to *Chaetozone* sp. MG8 in Grosse et al. (2020). Genuine *C. setosa* sequences from Svalbard and the White Sea are in currently in BOLD:AAB5820. The most similar bin, BOLD:AAB5819, is 4.8 % dissimilar and contains two clusters named *C. setosa* from Nunavut and British Columbia (Hardy et al., 2010; Carr et al., 2011). Three additional of our OTUs where returned with 100 % match to private sequences from the White Sea, also identified as *C. setosa.* However, these sequences do not cluster with any other *Chaetozone*. We suspect they are misidentified morphologically, but treat them provisionally as *Chaetozone WS* in this paper. Another OTU is matching Pacific sequences filed as *Chaetozone* sp. They cluster as sisters to *Tharyx* sp. in BOLD:AAB0838 (Carr et al., 2011; Hardy et al., 2010) and perhaps may refer to one of the Arctic species described by Blake (2015). Yet another set of “*C. setosa*” sequences in BOLD are collections from Shandong, China. We suspect they may perhaps be misidentified *Aphelochaeta.*

A recent revision (Meißner et al., 2020) points out that several CO1 barcodes assigned to *Pholoe baltica* in public databases are actually *P. assimilis*. Our BOLD search returned the latter identification, but GenBank matched the former misidentification.

The 100% similarity hit on *Cossura pygodactylata* refers to two private sequences from the Arctic. However, the confusion of *C. pygodactylata* and *C. longocirrata* may also have affected barcodes in BOLD, because the species do not cluster monophyletically*.*

Our sequences that gave 100 % match to Alaskan *Leitoscoloplos pugettensis* CMC03 (Carr et al., 2011; Hardy et al., 2010) may be misidentified because sequences with the same name are in several different clusters and BOLD appears to have a mixed complex of *Scoloplos* and *Leitoscoloplos* species that need to be critically revised.

Our identification of the bivalve *Serripes groenlandicus* is matching sequences in BOLD:ADO0464, which also contains N. Pacific *Serripes laperousii* (Layton et al., 2014). This warrants further examination of the relationship between the two. It should be noted that the sequences in this bin are short. Other *Serripes groenlandicus* are in two different bins, BOLD:AAH9554 and BOLD:AAH9554. A third Arctic species, *Serripes notabilis*, has no labelled sequences. We also detected the bivalve *Adontorhina* (Barry & Mccormack, 2007), a species that was originally barcoded from specimens collected by Mareano at Svalbard.

Matching sequences of the bivalves *Yoldiella frigida* and *Yoldiella nana* refer to specimens barcoded for BOLD by the University Museum of Bergen. The former species is in bin BOLD:AAO3807 and vouchers are from Svalbard. The bin also holds specimens from the Canadian Arctic identified to *Y. nana.* We suspect this is a misidentification. Our *Y. nana* references are from N. Atlantic and the sequences are in bin BOLD:AEN6973.

*Strongylocentrotus droebachiensis* and *S. pallidus* (Echinoidea) were both DNA-detected, the latter in both fjords studied. The two species may be difficult to separate morphologically (Gagnon & Gilkinson, 1994) and have complex genetics (Norderhaug et al., 2016), although the Folmer region of CO1 seems to separate these species.

Nematodes are rarely identified in standard ecosystem surveys (Dell’Anno et al., 2015; Derycke et al., 2010) and metabarcoding has opened for such opportunities. Whereas van den Heuvel‑Greve et al. (2021) recorded 33 taxa of nematodes from Kongsfjord based on 18S amplicons, Somerfield et al. (2006) recorded 110 putative species from morphology. Our CO1 sequencing only identified two species, *Terschellingia longicaudata and* *Parasphaerolaimus paradoxus*. *T. longicaudata* is considered as a more or less cosmopolitan complex of cryptic species (Armenteros et al., 2009; Bhadury et al., 2008; Sahraean et al., 2017). *Terschellingia distalamphida* has been recorded from the North Atlantic (Portnova, 2009) and *Terschellingia communis* additionally from Kongsfjord (Aswathy et al., 2017). It is presently unclear how these records correspond with known CO1 sequences (Sahraean et al., 2017) and 18S sequences (Bhadury et al., 2008; van den Heuvel-Greve et al., 2021). *P. paradoxus* was originally described from the North Atlantic (Zograf et al., 2017), but it is somewhat disturbing that our DNA identification is based on a 95 % similarity match with specimens that were collected in the Yellow Sea. A potential candidate species for Svalbard waters is *Parasphaerolaimus polaris* (Zograf et al., 2017). However, the species is not represented with sequences in BOLD.

The hydroid *Lafoea dumosa* occur in three bins, of which one is a cluster of East Pacific specimens.

BOLD hit suggests *Halcampa decemtentaculata (*Anthozoa*)* for the sequences matching *Halcampa chrysanthellum* in GenBank. Morphological identifications by the Mareano team have recorded *Halcampa arctica* in these waters, but the species has not been barcoded. *H. decemtentaculata* is recorded in WORMS (2012-06-12) as a Pacific species whilst the other two are Arctic Atlantic*.* We provisionally handle this identification as *Halcampa* sp.

According to Miranda et al. (2016), the cnidarian *Lucernaria bathyphila* is paraphyletic and needs critical systematic revision. Our sequences cluster with *Lucernaria janetae* and a sequence from the Faroe Islands. *Lucernia quadricornis* has been recorded from the region, but CO1 barcodes of the species have not been defined.

It seems likely that our Bold hit on *Plicatellopsis fragilis* (Porifera) is actually *Plicatellopsis bowerbanki*. The former is known from Antarctica. The latter is Arctic and has its type locality at the North Cape Bank. Blastn identified the amplicons to *Phakellia bowerbanki*, which is currently an unaccepted name (van Soest et al., 2021).

Additional references (to Taxonomic remarks)

Armenteros M, Ruiz-Abierno A, Vincx M, Decraemer W. 2009 A morphometric analysis of the genus *Terschellingia* (Nematoda: Linhomoeidae) with redefinition of the genus and key to the species. *Journal of the Marine Biological Association of the United Kingdom*. Cambridge University Press 89(6):1257–67. <https://doi.org/10.1017/S0025315409000381>

Aswathy NK, Athira AT, Krishnapriya PP, Jima M, Akhilesh Vijay, Bijoy Nandan S, Krishnan KP. 2017 Functional trait analysis of meiobenthic nematodes in the Arctic Kongsfjord. *Journal of Aquatic Biology & Fisheries* 5:65-78.

Bhadury P, Austen MC, Bilton DT, Lambshead PJD, Rogers AD, Smerdon GR. 2008. Evaluation of combined morphological and molecular techniques for marine nematode (*Terschellingia* spp.) identification. *Marine Biology* 154: 509–518. <https://doi.org/10.1007/s00227-008-0945-8>.

Barry PJ, Mccormack G. 2007. Two new species of *Adontorhina* Berry, 1947 (Bivalvia: Thyasiridae) from the Porcupine Bank, off the west coast of Ireland. *Zootaxa* 1526(1):37. <https://doi.org/10.11646/zootaxa.1526.1.2>

Blake JA. 2015. New species of *Chaetozone* and *Tharyx* (Polychaeta: Cirratulidae) from the Alaskan and Canadian Arctic and the Northeastern Pacific, including a description of the lectotype of *Chaetozone setosa* Malmgren from Spitsbergen in the Norwegian Arctic. *Zootaxa* 3919 (3):501–552. <https://doi.org/10.11646/zootaxa.3919.3.5>

Borja A, Franco J, Pérez V. 2000. A Marine Biotic Index to Establish the Ecological Quality of Soft-Bottom Benthos Within European Estuarine and Coastal Environments. *Marine Pollution Bulletin* 40(12):1100-1114.

<https://doi.org/10.1016/S0025-326X(00)00061-8>.

Carr CM, Hardy SM, Brown TM, Macdonald TA, Hebert PD. 2011. A tri-oceanic perspective: DNA barcoding reveals geographic structure and cryptic diversity in Canadian polychaetes. *PLoS One* 6(7):22232. <https://doi.org/10.1371/journal.pone.0022232>.

Derycke S, Vanaverbeke J, Rigaux A, Backeljau T, Moens T. 2010 Exploring the use of cytochrome oxidase c subunit 1 (COI) for DNA barcoding of free-living marine nematodes. *PLOS One* 5:e13716. <https://doi.org/10.1371/journal.pone.0013716>

Gagnon J-M, Gilkinson KD. 1994. Discrimination and distribution of the sea urchins *Strongylocentrotus droebachiensis* (O.F. MULLER) and *S. pallidus* (G.O. SARS) in the Northwest Atlantic. *Sarsia* 79:1-11. <https://doi.org/10.1080/00364827.1994.10413542>.

Grosse M, Bakken T, Nygren A, Kongsrud JA, Capa M. 2020 Species delimitation analyses of NE Atlantic *Chaetozone* (Annelida, Cirratulidae) reveals hidden diversity among a common and abundant marine annelid. *Molecular Phylogenetics and Evolution* 149:106852. <https://doi.org/10.1016/j.ympev.2020.106852>.

Grosse M, Capa M, Bakken T. 2021. Describing the hidden species diversity of *Chaetozone* (Annelida, Cirratulidae) in the Norwegian Sea using morphological and molecular diagnostics. *ZooKeys* 1039:139–176. <https://doi.org/10.3897/zookeys.1039.61098>.

Hardy SM, Carr CM, Hardman M, Steinke D, Corstorphine E, Mah C. 2010. Biodiversity and phylogeography of Arctic marine fauna: insights from molecular tools. *Marine Biodiversity* 41(1):195-210. <https://doi.org/10.1007/s12526-010-0056-x>.

Layton KK, Martel AL, Hebert PD 2014. Patterns of DNA Barcode Variation in Canadian Marine Molluscs. *PLoS ONE* 9(4):e95003. <https://doi.org/10.1371/journal.pone.0095003>

Meißner K, Götting M, Nygren A. 2019. Do we know who they are? On the identity of *Pholoe* (Annelida: Sigalionidae: Pholoinae) species from northern Europe. *Zoological Journal of the Linnean Society* 189:178–206. <https://doi.org/10.1093/zoolinnean/zlz120>

Miranda LS, Hirano YM, Mills CE, Falconer A, Fenwick D, Marques AC, Collins AG. 2016. Systematics of stalked jellyfishes (Cnidaria: Staurozoa). *PeerJ* 4:e1951. <https://doi.org/10.7717/peerj.1951>.

Norderhaug KM, Angles d'Auriac MB, Fagerli CW, Gundersen H, Christie H, Dahl K, Hobæk A. 2016. Genetic diversity of the NE Atlantic sea urchin *Strongylocentrotus droebachiensis* unveils chaotic genetic patchiness possibly linked to local selective pressure. *Marine Biology* 163,36. <https://doi.org/10.1007/s00227-015-2801-y>

Portnova D. 2009. Free-living nematodes from the deep-sea Håkon Mosby Mud Volcano, including the description of two new and three known species. Zootaxa 2096:197–213. <https://doi.org/10.11646/ZOOTAXA.2096.1.13>.

Sahraean N, van Campenhout J, Rigaux A, Mosallanejad H, Leliaert F, Moens T. 2017. Lack of population genetic structure in the marine nematodes *Ptycholaimellus pandispiculatus* and *T. longicaudata* in beaches of the Persian Gulf, Iran. *Marine Ecology* 38:e12426. . <https://doi.org/10.1111/maec.12426>

van Soest RWM, Boury-Esnault N, Hooper JNA, Rützler K, de Voogd NJ, Alvarez B, Hajdu E, Pisera AB, Manconi R, Schönberg C, Klautau M, Kelly M, Vacelet J, Dohrmann M, Díaz M-C, Cárdenas P, Carballo JL, Ríos P, Downey R, Morrow CC. 2021. World Porifera Database. *Phakellia bowerbanki* Vosmaer, 1885. *Accessed through*: World Register of Marine Species at: [http://www.marinespecies.org/aphia.php?p=taxdetails&id=132506 on 2021-05-01](http://www.marinespecies.org/aphia.php?p=taxdetails&id=132506%20on%202021-05-01)

Zograf JK, Pavlyuk ON, Trebukhova YA, Tu DN. 2017. Revision of the genus *Parasphaerolaimus* (Nematoda: Sphaerolaimidae with description of new species. *Zootaxa* 4232(1):058–070. <https://doi.org/10.11646/zootaxa.4232.1.4>.
